# Intestinal flora metabolites indole-3-butyric acid and disodium succinate promote IncI2 *mcr-1-*carrying plasmid transfer

**DOI:** 10.3389/fcimb.2025.1564810

**Published:** 2025-06-03

**Authors:** Jialiang Xu, Mengke Zhang, Yi Yan, Zhe Li, Xin Lu

**Affiliations:** ^1^ School of Light Industry Science and Engineering, Beijing Technology and Business University, Beijing, China; ^2^ National Key Laboratory of Intelligent Tracking and Forecasting for Infectious Diseases, National Institute for Communicable Disease Control and Prevention, Chinese Center for Disease Control and Prevention, Beijing, China

**Keywords:** *mcr-1*-carrying plasmid, intestinal flora metabolite, conjugation, IncI2, IBA, DS

## Abstract

**Introduction:**

Plasmid-driven horizontal transfer of resistance genes in bacterial communities is a major factor in the spread of resistance worldwide. The gut microbiome, teeming with billions of microorganisms, serves as a reservoir for resistance genes. The metabolites of gut microorganisms strongly influence the physiology of their microbial community, but the role of the metabolites in the transfer of resistance genes remains unclear.

**Methods:**

A dual-fluorescence conjugation model was established. We assessed the effects of different concentrations of indole-3-butyric acid (IBA) and disodium succinate (DS) on plasmid transfer using conjugation assays. The growth of bacteria (donors, recipients, and transconjugants), the reactive oxygen species (ROS) levels and membrane permeability were measured under IBA and DS exposure. The plasmid copy number, and transcriptional levels of conjugation-related genes (including the related genes of the regulation of ROS production, the SOS response, cell membrane permeability, pilus generation, ATP synthesis, and the type IV secretion system (T4SS) ) were evaluated by qPCR.

**Results:**

In this study, we demonstrated that IBA and DS at low concentrations, which can also be ingested through diet, enhance the interspecies transfer ratio of IncI2 *mcr-1*-carrying plasmid in *Escherichia coli*. At 20 mg/L, the transfer ratios in the presence of IBA or DS increased by 2.5- and 2.7-fold compared to that of the control, respectively. Exposure to this concentration of IBA or DS increased the production of reactive oxygen species (ROS), the SOS response, cell membrane permeability, and plasmid copy number. The transcription of genes of the related pathways and of pilus, ATP, and the T4SS was upregulated.

**Discussion:**

Our findings revealed that low-dose gut microbiota metabolites—particularly those with dietary origins—promote plasmid-mediated resistance gene dissemination through multifaceted mechanisms involving oxidative stress, SOS activation, and conjugation machinery enhancement. This highlights potential public health risks associated with microbiota metabolites, especially those utilized in food production.

## 1 Introduction

Plasmid-mediated horizontal transfer of resistance genes is the primary means of resistance spread. The discovery of mobile colistin resistance genes (*mcr*) on plasmids changed the view that polymyxin resistance was solely caused by chromosomal gene mutations or regulation, and implied higher risks of dissemination and increased clinical treatment costs (Liu et al., 2016). Over the years, the *mcr-1* carrying plasmid (pMCR-1) was found to be transmitted among animals, humans, and food in different environments in more than seventy countries on six continents (Mmatli et al., 2022). Epidemiological studies indicated that almost all colistin resistance in *Escherichia coli* is mediated by the *mcr* genes, and the increase in colistin resistance is attributed to the dissemination of the *mcr-1* gene (Wang et al., 2020; Shen et al., 2020, Liu et al., 2020). The IncI2 plasmid is considered the optimal vector for *mcr-1* transmission and represents the first reported replicon type of *mcr-1* plasmids. Its structure is conserved, and it can carry co-transfer carbapenemases-producing genes or extended-spectrum β-lactamases-producing genes (Meinersmann, 2019). Therefore, IncI2 pMCR-1 is a good representative for studying polymyxin resistance dissemination patterns (Wilkinson et al., 2022).

The transfer of plasmids is influenced by various stress factors caused by the ecological niche of the host bacteria. A typical example is antibiotic selection pressure, in which sub-inhibitory concentrations of antibiotics facilitate plasmid transfer (Ortiz et al., 2021). Similarly, a recent study has found that non-antibiotic chemicals in the environment, such as heavy metals (Zhao et al., 2019), disinfectants (Guo et al., 2015), non-antibiotic pharmaceuticals (Yang et al., 2022), CO_2_ (Liao et al., 2019), nonnutritive sweeteners (Yu et al., 2021), petrol and diesel exhaust particles (Zhang et al., 2018) facilitated the horizontal transfer of antibiotic resistance genes (ARGs). The risk factors that promote antibiotic-resistance plasmid transfer in bacterial environments are receiving increasing attention.

The intestinal microbiota is a critical reservoir for storing and disseminating resistance plasmids. The vast array of metabolites produced by the microbiota are directly implicated in human health, including those participating in nutrient metabolism (Mithul et al., 2021), drug metabolism (Pant et al., 2023), maintenance of the structural integrity of the intestinal mucosal barrier (Recharla et al., 2023), immune regulation (Liu et al., 2021) and resistance to pathogens (Beaud et al., 2005). It is noteworthy that intestinal flora metabolites also act as microenvironmental stress factors to influence the transfer of resistance plasmids, however, there are scant reports on this. Short-chain fatty acids can alter intestinal pH, potentially changing the physiological state of the bacteria and indirectly inhibiting the transfer of resistance plasmids among intestinal microbes (Ott and Mellata, 2024). We focused on the effects of metabolites on the transfer of resistance plasmids by screening 172 intestinal microbial metabolites. We ultimately observed that low concentrations of indole-3-butyric acid (IBA) and disodium succinate (DS) promoted IncI2 pMCR-1 transfer. Specifically, IBA was produced by the opportunistic pathogen *Clostridium difficile* (Brooks et al., 1984), which is frequently used in agriculture to enhance plant growth (Frick and Strader, 2018). DS, produced by *Lactobacillus* generating succinic acid, combines with sodium ions (Tsuji et al., 2013), to serve as a food additive that imparts an umami flavor to various food products (Ma et al., 2020). Both IBA and DS can ingest through the diet (Tian et al., 2022). Recent research suggested that specific ingredients found in food and oral medication, such as paclitaxel (Yang et al., 2022) and artificial sweeteners (Yu et al., 2023) may promote the transfer of resistance plasmids. Currently, the study of the risks posed by metabolites in the gut, including the spread of antibiotic resistance, remains a blind spot but cannot be overlooked, particularly the risks of metabolites that are used as food additives.

Current studies showed that the increased transfer ratio of resistance plasmids was associated with changes in multiple regulatory pathways. Nonnutritive sweeteners were shown to promote plasmid transfer by upregulating reactive oxygen species (ROS) generation, the SOS response, and cell membrane permeability (Yu et al., 2021). A diet high in fat, sugar, and salt promoted the expansion and transfer of exogenous ARGs in gut microbes, while significantly changing the gut microbiota and its metabolic product composition. This may be attributed to a SOS response or to changes in bacterial membrane permeability, bacterial composition and bacterial diversity due to diet-induced inflammation (Tan et al., 2022). The previous research reported that the cell membrane permeability and conjugative transfer-related genes were modulated by overproduction of ROS and reactive nitrogen species during bacterial exposure to glyphosate, increasing the conjugative transfer ratio of ARGs (Zhang et al., 2021). Plasmid-encoded the type IV secretion system (T4SS) is an essential macromolecular apparatus for conjugation, and exposure to sulfonamide antibiotics significantly upregulated T4SS-related genes expression, thereby enhancing transfer ratio (Zhao et al., 2021). Cyromazine and kresoxim-methyl, which stimulated bacterial ATP production, enhanced the conjugation process energy supply (Zhu et al., 2024). The transfer of a plasmid is co-regulated by functional pathways encoded by the host bacteria and by the plasmid itself, the novel mechanisms of this sensitive and complex process remain to be further explored.

In this study, we observed that low concentrations of IBA or DS promoted IncI2 pMCR-1 transfer. Respectively, increased ROS production and cell membrane permeability were observed after exposure to IBA or DS and were verified at the transcription level. In addition, the core genes of related functional pathways, such as ATP synthesis, pilus generation, and T4SS, were upregulated. This study underscored an unrecognized function of intestinal metabolites of accelerating the spread of drug resistance, which is particularly concerning given that some of these metabolites are also used in the food industry. These findings served as a reminder of the potential double-edged sword effects of these metabolites, which should not be overlooked in food production applications.

## 2 Materials and methods

### 2.1 Bacteria strains, culture conditions, and metabolic substrates

The donor of the conjugation model was *E. coli* MG1655 with resistance to rifampicin (Rfp^R^) carrying the plasmid pSH13G841 containing the *mcr-1* gene. The plasmid pSH13G841 was isolated from *Salmonella* Typhimurium obtained from fecal samples of a 9-month-old male community-acquired diarrhea patient in Shanghai (Lu et al., 2019). The recipient strain was *E. coli* CNE6, isolated from fecal samples.

The strains were grown in liquid medium (Luria-Bertani) or on medium containing 1.5% agar with corresponding antibiotics added (100 μg/mL rifampicin, 100 μg/mL streptomycin, 4 μg/mL polymyxin B). Single colonies were selected and incubated with shaking at 180 rpm for 18 h at 37°C. Transfer the culture to fresh medium at a 1:1000 ratio until it reaches the log phase (OD_600_ = 0.5), the cells were washed twice by centrifuging at 3000 rpm for 5 min and resuspending to an OD_600_ of 0.75 for conjugation transfer.

Indole-3-butyric acid (IBA, C12H13NO2, CAS133-32-4) and disodium succinate (DS, C4H14Na2O4, CAS150-90-3) with purity > 98% were purchased from Macklin and prepared as a concentrated stock solution in dimethyl sulfoxide (DMSO, Biotopped) and sterile ultra-pure water at 0.016, 0.08, 0.4, 2, 10, 30 and 50 mg/mL, respectively. All solvents and reagents used were of analytical grade. The final concentration of IBA or DS used in the experimental system was both one hundredth of that of the concentrated stock solution.

### 2.2 Induction of streptomycin resistance in the CNE6

The streptomycin resistant CNE6 strain was obtained through induction with a gradient of streptomycin concentrations pressure, and the chromosomal mutations occurred on the streptomycin resistance gene 16S rRNA. Specifically, single colonies were selected and incubated into liquid medium without antibiotics at 180 rpm for 18 h at 37°C. 100 μL of bacterial suspension was plated on medium containing 50 mg/L streptomycin and incubated at 37°C overnight. Subsequently, single colonies were selected and incubated into liquid medium without antibiotics at 180 rpm for 18 h at 37°C. Then, 100 μL of bacterial suspension was plated on medium containing 100 mg/L streptomycin and incubated at 37°C overnight. As described above, bacterial suspension was sequentially plated on medium containing 200 mg/L and 500 mg/L streptomycin and incubated at 37°C overnight. Single colonies were finally selected from medium containing 500 mg/L streptomycin for subsequent experiments.

### 2.3 Dual-fluorescence reporter system

To rapidly achieve screening of the transconjugants, we constructed a dual fluorescence system by using the suicide plasmid-mediated homologous recombination to insert the *mCherry* gene into the MG1655 chromosomal genome, and insert *gfp* gene into the resistance plasmid pSH13G841 genome. Using pWM91/pWM91-Cm as the suicide plasmid (Metcalf et al., 1996), an approximately 1000-bp fragment of the insertion site was selected as the homologous arms for recombination. The fluorescent reporter genes were linked to the constitutive promoter PLtetO-1 between the upstream and downstream homology arms by overlap PCR. The reconstituted fragments were mixed with the linearized pWM91/pWM91-Cm vector by seamless cloning (pEASY-Seamless Cloning and Assembly Kit, TransGen Biotech) and transferred into *E. coli* SM10λpir. The suicide plasmid carrying the homologous arms were transferred into the recipient bacteria (MG1655/J53-pSH13G841) by conjugation. After the transconjugants were selected on plates containing dual antibiotics, they were incubated on a medium containing 10% sucrose at 22°C for 72 h, the strains sensitive to ampicillin were selected for PCR validation and confirmed by sequencing. Based on this approach, we inserted the fluorescent reporter gene *gfp* between 18,643-18,644 bp of the plasmid (MH522411.1), resulting in the construction of the green fluorescent plasmid pSH13G841. We inserted *mCherry* between *aslA* and *aslB* in MG1655, thereby the host bacterium MG1655 with the red fluorescent was constructed. To form the donor for metabolite screening, the plasmid pSH13G841 with the green fluorescence was transferred into MG1655 with the red fluorescent. The dual-fluorescence reporter system MG1655::*mCherry*/pSH13G841-*gfp* was ultimately generated. All plasmids, primers and strains information were provided in the **Supplementary Tables S1** and **S2**.

### 2.4 Construction of the conjugation transfer experiment

The donor strain MG1655::*mCherry*/pSH13G841-*gfp* and recipient CNE6 were mixed in a 1:1 ratio (OD_600_ = 0.75, 180 μL) and subjected to static conjugation at 37°C. To clarify the conjugative time required for maximal transfer ratio achievement, multiple conjugation intervals (6, 12, 18, and 24 h) were established. The mixtures were plated on two selective medium: a single-antibiotic medium containing 100 mg/L streptomycin for the selection of recipients, and a double-antibiotic medium containing 100 mg/L streptomycin and 4 mg/L polymyxin B for the selection of transconjugants. The strains were confirmed by observation through fluorescence microscopy. Plasmid transfer ratio (f) was calculated as f=N(T)/N(R) , where N(T) and N(R) represented the numbers of transconjugants and recipients, respectively. We determined that the optimal conjugative time was 18 h when the transfer ratio reaches its maximum value (**Supplementary Figure S1**).

### 2.5 Collection and screening of intestinal flora metabolites

In this study, the Gut Microbial Metabolite Library (MedChemExpress, USA, Cat. No.: HY-L078), which including 172 intestinal flora metabolites, were purchased in 2024 and used for this research.

The final concentration of the intestinal flora metabolites, which was used in the conjugation experiment, was obtained by diluting the commercialized concentration at a ratio of 1:100 (**Supplementary Table S4**). Based on the dual-fluorescence reporter system (method 2.3) and conjugation experiment (method 2.4), for the metabolite-treated group, we introduced 1.8 μL each intestinal flora metabolite during the static donor and recipient strain conjugation phase, and calculated the conjugative transfer ratio. We constructed three control groups based on the different types of solvents (DMSO/sterile water/anhydrous ethanol) contained in each metabolite. For the control group, 1.8 μL DMSO/sterile water/anhydrous ethanol was added during the static donor and recipient strain conjugation phase and calculated the conjugative transfer ratio, respectively. The effect of intestinal flora metabolites on plasmid transfer was assessed by the ratio of the conjugative transfer ratio between the metabolite-treated group and the control group. Metabolite with the ratio greater than 2.5 was considered to have significant effect on plasmid transfer.

### 2.6 Conjugation and bacterial growth under IBA and DS exposure

The same conjugation experiments were established as described above and the mixture of recipient and donor was exposed to different concentrations (0.16, 0.8, 4, 20, 100, 300, 500 mg/L) of IBA and DS during conjugation, respectively. DMSO and sterile ultra-pure water were mixed separately in equal volumes as control groups, and the conjugative transfer ratios were calculated.

In addition, adding ROS scavenger (N-acetyl-L-cysteine, 100 μM, Yeasen Biotechnology) in the conjugation experiments to determine whether IBA or DS promoted conjugative transfer via ROS generation. The conjugative transfer ratios of the ROS scavenger-treated group were then compared with that of the non-ROS scavenger-treated group (Lu et al., 2018).

The growth curves of recipients, donors, and transconjugants which obtained under the different concentrations of IBA or DS treated group were generated to analyze their growth (Wang et al., 2019). The donors, recipients, and transconjugants were cultivated at 37°C and 180 rpm for 18 h, and then inoculated into fresh medium at a ratio of 1:1000. Subsequently, 200 μL samples were added to a 100-well microplate, and the OD_600_ values were measured every 2 h at 37°C using the automated growth curve analyzer (Bioscreen C, Bioscreen, Finland). All experiments were conducted with a minimum of three biological replicates.

To further verify that IBA promoted the transfer of resistance plasmids, we conducted conjugation experiments. The mixture of donor and recipient bacteria were exposed to low concentration of IBA (4–100 mg/L), and were statically incubated at 37°C for 14, 15, 16, 17, and 18 h, respectively. The conjugative transfer ratio was calculated using the previously described method. Furthermore, the growth states for the donor, recipient, and transconjugant in 20 mg/L IBA treatment group were assessed, using the same measurement protocol as previously established.

### 2.7 Measurement of ROS

2’,7’-Dichlorofluorescein diacetate (DCFH-DA, MedChemExpress, USA) was used for intracellular ROS generation (Lu et al., 2018). Briefly, both the donor MG1655 and recipient CNE6 cell suspensions were incubated individually with 10 μM of DCFH-DA in the dark for 30 min at 37°C on a 100 rpm shaker. The bacterial suspensions were washed with PBS to remove the extracellular DCFH-DA probe, and treated with different concentrations of IBA or DS for 2 h at 37°C in the dark. The samples were then transferred into a black 96-well plate, 200 μL per well, to measure the fluorescence intensity (excitation 488 nm, emission 525 nm) with a microplate reader (EnSight, PerkinElmer, USA). The level of intracellular ROS generation was evaluated by the normalized fluorescence intensity value of the treated samples to that of the controls. Additionally, to further verify that ROS production was induced by both IBA and DS, 100 μM of N-acetyl-L-cysteine (a scavenger of oxygen free radicals) was added to the same sets of the samples as described above, and the ROS levels of the ROS scavenger-treated group were compared with that of the non-ROS scavenger-treated group (Lu et al., 2018).

### 2.8 Measurement of cell membrane permeability

The propidium iodide fluorescence (PI, Yeasen Biotechnology, China) labeling method was used to measure the membrane permeability of the donor and recipient strains exposed to IBA or DS, respectively (He et al., 2022). Specifically, after culturing at 37°C and 200 rpm for 18 h, the cultures were transferred to fresh LB and incubated until the OD_600_ was 0.5. The donor and recipient bacteria were washed twice with PBS, resuspended, and exposed to different concentrations of IBA or DS at 37°C for 2 h, respectively. A final concentration of 5 mg/L of PI was added to the PBS with the suspended cells and incubated at 37°C for 30 min in the dark with shaking at 100 rpm. Next, the samples were transferred into the black 96-well plates to measure the fluorescence intensity (excitation 488 nm, emission 630 nm) with a microplate reader (EnSight, PerkinElmer, USA). Untreated and heat-treated (2 h at 80°C) samples were used as the controls for intact and damaged cells, respectively.

### 2.9 Determination of plasmid copy number

The conjugation systems were established under the IBA or DS concentrations of 4, 20, and 100 mg/L, respectively. In brief, the donor and recipient were mixed in 1:1 ratio and exposed to different concentrations of IBA or DS at 37°C for 18 h. Then, the 2 mL of bacterial suspension was collected and nucleic acids were extracted (TIANamp Genomic DNA Kit, TIANGEN). Based on previous research, the target gene *mcr-1* was analyzed using real-time quantitative fluorescence PCR detection (Zhang et al., 2021). The qPCR mixtures consisted of 10 μL 2×SYBR Green mix, 0.4 μL forward primers, 0.4 μL reverse primers, 1 μL DNA, and 7.2 μL sterile H_2_O. The total qPCR volume was 20 μL. The program for qPCR was as follows: a 95°C predenaturation for 15 min, 45 cycles of denaturation at 95°C for 30 s, annealing at 60°C for 60 s, and extension at 72°C for 30 s. Subsequently, using *recA* as the reference gene, the expression of the *mcr-1* gene was measured using a qPCR (Gentier96E, TIANLONG, China) to reflect the copy number of the resistance plasmid.

### 2.10 Determination of transcription levels of conjugation-associated regulatory genes

The mRNA was respectively extracted from the conjugation system and donor at a concentration of 20 mg/L of IBA or DS treated group. In brief, a conjugation system was established by mixing the donor and recipient strains at a 1:1 ratio, and then the conjugation system and donor strain were exposed to IBA or DS at 37°C for 2 h, respectively. The bacteria were collected by centrifugation at 3000 rpm at 4°C for 5 min. The total RNA was extracted using the Trizol method (Rio et al., 2010). After ensuring the quality of the mRNA by electrophoresis, the reverse transcription was performed with a reverse transcription kit (SuperScript™ III reverse transcriptase, Thermo Fisher). Using *recA* as the reference gene, qPCR was performed to measure the transcription levels of genes (including the related genes of the ROS detoxification, the SOS response, cell membrane permeability, pilus generation, ATP synthesis, and the T4SS). All the target gene primers were evaluated by the efficiency of their amplification, and their information is provided in **Supplementary Table S3**.

### 2.11 Statistical analysis

All statistical analyses in this study were performed using GraphPad Prism 9.0. For the two-sample unpaired *t*-tests, two-tailed *p*-values and 95% confidence intervals were used. If the data variance was unequal, Welch’s correction was applied. If the data did not follow a normal distribution, the Mann–Whitney test, a nonparametric test, was used. *p*-values < 0.05, *p*-values < 0.01, and *p*-values < 0.001 were defined as significant differences.

## 3 Results

### 3.1 Low concentrations of IBA or DS promoted IncI2 pMCR-1 conjugative transfer

**Figure 1:**
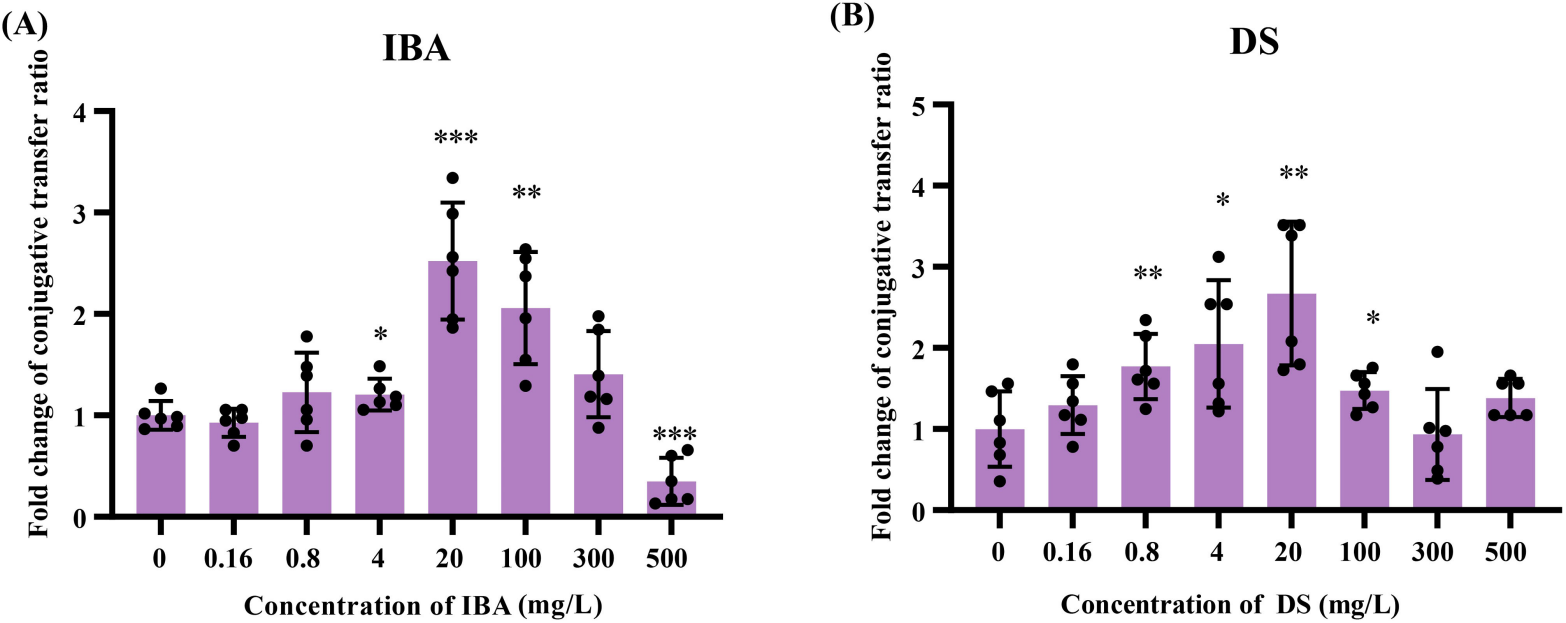
The effects of IBA and DS on the transfer ratios of IncI2 pMCR-1 in *E*. *coli* conjugation pairs. Fold changes in plasmid transfer ratios after 18 h of IBA **(A)** and DS **(B)** treatment were shown. The results represent the mean ± SD of six biological samples. Significant differences between the IBA or DS treatment groups at the different concentrations and the control group were tested by *t*-test and indicated by *p < 0.05, **p < 0.01, and ***p < 0.001.

We evaluated the effects of each metabolite from the Gut Microbial Metabolite Library (including 172 intestinal flora metabolites) on the transfer of the IncI2 *mcr-1*-carrying plasmid (pMCR-1) and identified two metabolites, IBA and DS, that significantly promoted plasmid transfer, with fold changes of 2.5 and 2.8 (**Supplementary Table S4**), respectively. We confirmed that low concentrations (4–100 mg/L) of IBA or DS can promote the transfer of the IncI2 pMCR-1 between *E. coli* strains, respectively (**Figure 1**, **Supplementary Figure S2**). We observed the dose-dependent effect between the plasmid transfer ratio and the concentrations of IBA/DS, in which the transfer ratios first increased and then decreased with the concentrations of IBA/DS increased.

The low concentrations (4–100 mg/L) of IBA or DS significantly promoted the conjugative transfer of IncI2 plasmids, and the maximum conjugative transfer ratio was showed at 20 mg/L, increasing by 2.5-fold (IBA, **Figure 1A**) and 2.7-fold (DS, **Figure 1B**), respectively, over that of the control group. When the concentration of IBA was 500 mg/L, the conjugative transfer ratio in the IBA-treated group significantly decreased to 0.37-fold that of the control group (*p* < 0.05). In contrast, the conjugative transfer ratios in the DS-treated group returned to a level comparable to that of the control group. Additionally, the absolute transfer ratio was presented in **Supplementary Figure S2**.

**Figure 2:**
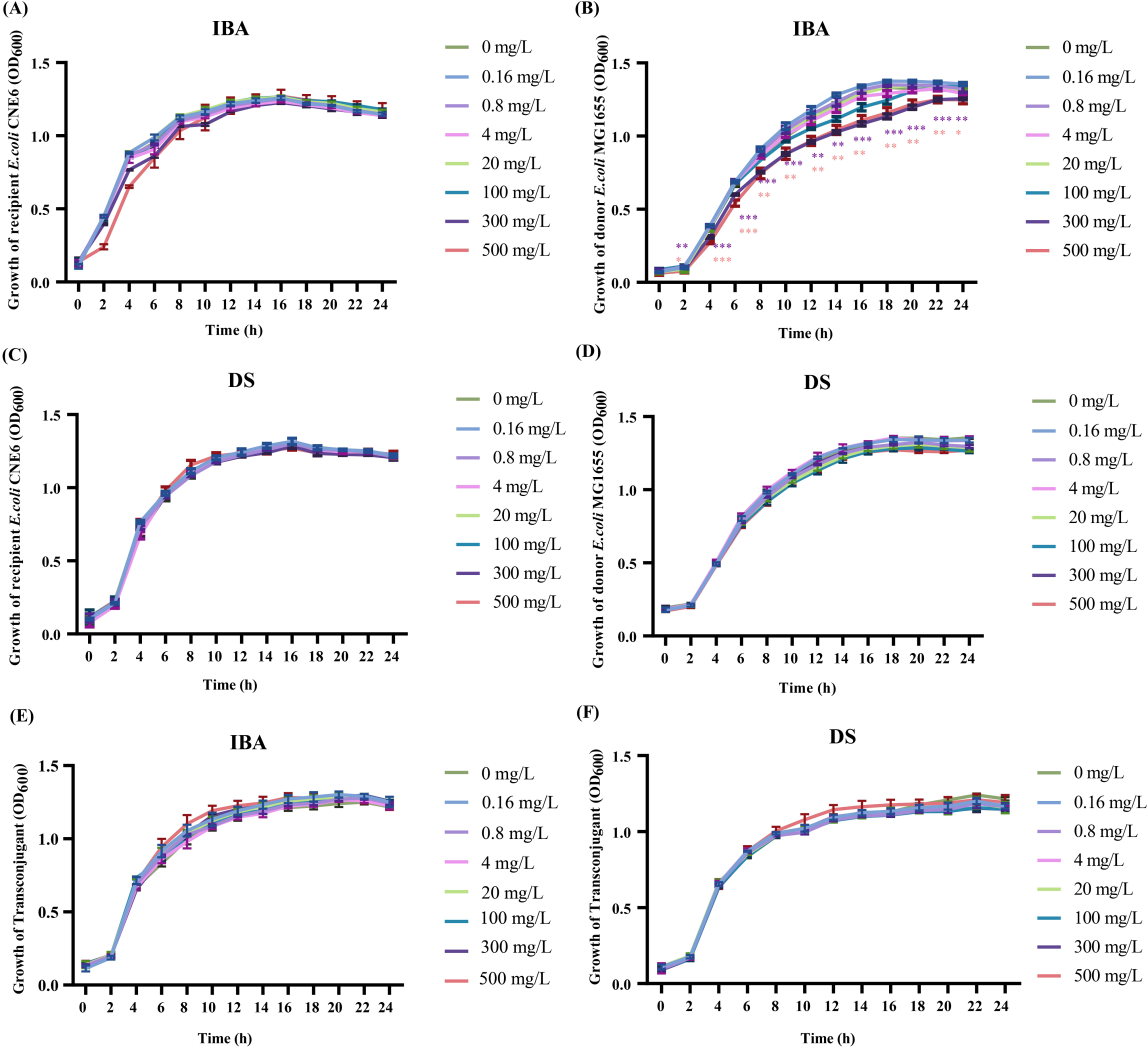
The effects of IBA and DS on the growth of donor, recipient, transconjugant. The growth situation of the recipient, donor and transconjugant with IBA **(A, B, E)** and DS **(C, D, F)** were shown. The results represent the mean ± SD of three biological samples. Significant differences between the IBA or DS treatment groups at the different concentrations and the control group were tested by *t*-test and indicated by *p < 0.05, **p < 0.01, and ***p < 0.001.

The metabolites can affect the growth of donors and recipients, manifesting as changes in the conjugation ratio. The OD_600_ value was measured to represent the growth of the donor, recipient, and transconjugant (**Figure 2**). Low concentrations (0.16–20 mg/L) of IBA or DS had no significant effect on either the viability of donor, the recipient, or the transconjugant (**Figure 2**). However, 300 and 500 mg/L of IBA inhibited the growth of the donor (*p* < 0.05, **Figure 2B**).

Due to the high concentrations of IBA (300–500 mg/L) inhibiting the growth of donor, we investigated whether 20 mg/L IBA increase the conjugative transfer ratio of the IncI2 pMCR-1 by altering the growth states of the donor, recipient, or transconjugant. The result showed that 20 mg/L IBA increased the conjugative transfer ratio, with 18-hour incubation group demonstrating the highest transfer ratio (*p*<0.05, **Supplementary Figures S3A-E**). Additionally, in comparison with the control group, treatment with 20 mg/L IBA did not affect the growth states of the donor, recipient, or transconjugant (**Supplementary Figures S3F-H**). These results ruled out the possibility that 20 mg/L IBA promoted the transfer of the *mcr-1*-carrying IncI2-type plasmid by affecting the growth of the donor, recipient, and transconjugants. The raw data were provided in **Supplementary Tables S5**-**S8**.

### 3.2 Low concentrations of IBA or DS enhanced ROS production and the SOS response

**Figure 3:**
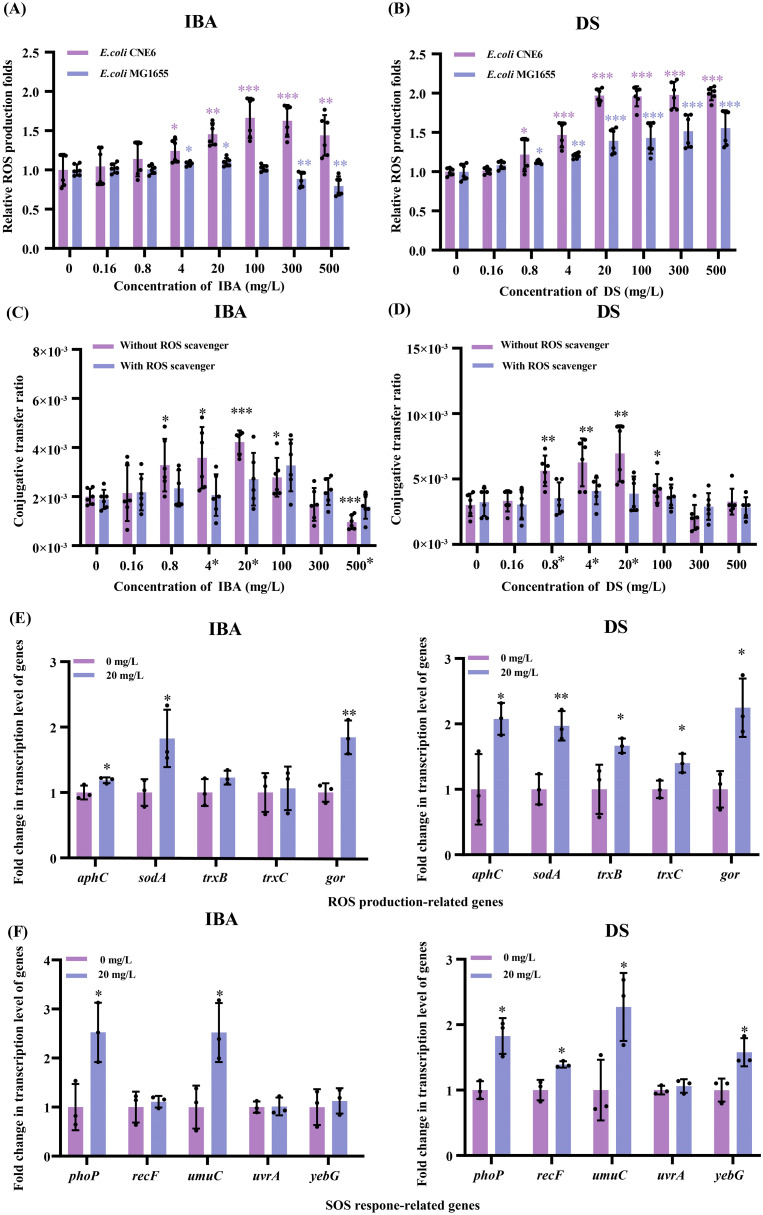
Changes related to the ROS production and the SOS respone after exposure to IBA and DS. Fold changes in ROS production by *E*. *coli* MG1655 and *E*. *coli* CNE6 under IBA **(A)** and DS **(B)** exposure for 2 h were shown. The ratios of conjugative transfer under IBA **(C)** and DS **(D)** in the presence and absence of the ROS scavenger were shown. The results represent the mean ± SD of six biological samples. The * on the numbers indicated inter-group differences, while the * in the graph represented intra-group differences. Fold changes in the transcription levels of ROS **(E)** and SOS **(F)** related genes in the conjugation systems were shown. The results represent the mean ± SD of three biological samples. Significant differences between the IBA or DS treatment groups at the different concentrations and the control group were tested with *t*-test and indicated by *p < 0.05, **p < 0.01, and ***p < 0.001.

Excessive ROS production triggers bacterial oxidative stress, which in turn facilitates the transfer of ARGs. Thus, we measured ROS production of the donor and recipient exposed to IBA or DS, respectively. In the IBA-treated group, the ROS levels of both the recipient and donor bacteria increased and then decreased as the IBA concentration increased. The donor and recipient bacteria exhibited their maximum levels of ROS at treatment concentrations of 20 mg/L and 100 mg/L, respectively, which corresponded to 1.10- and 1.66-fold over the control group ROS levels. More than 20 mg/L IBA, ROS levels of the donor bacteria began to decline with the IBA concentrations continued to increase, decreasing by one-fifth of the control group level at 500 mg/L IBA. In comparison, ROS levels in the donor and recipient bacteria in the DS-treated group continuously increased as the DS concentration increased, reaching a maximum at 500 mg/L DS, at 1.55- and 1.99-fold of the control group ROS levels, respectively (*p* < 0.05, **Figure 3B**).

The relationship between increased ROS levels and enhanced transfer was further confirmed by adding ROS scavengers to the conjugation system. We observed that the ROS levels of the donor and recipient returned to the levels of the control group after adding ROS scavengers (**Supplementary Figure S4**). The conjugative transfer ratio in the ROS scavenger group was restored to that of the control group (**Figures 3C, D**). This suggested that the increase in ROS production induced by low concentrations of IBA or DS contributed to the increase in the plasmid transfer ratio.

We compared the transcription levels of the genes related to ROS regulation in the conjugation systems with and without IBA/DS treatment (**Figure 3E**, **Supplementary Table S9**). In the IBA or DS treatment groups, the transcription of genes linked to the bacterial antioxidant defense system were significantly upregulated, respectively. Specifically, the expression of *aphC*, *sodA*, and *gor* genes increased by 1.19–1.84-fold (IBA) and 1.97–2.25-fold (DS) over that of the control. Following treatment with DS, the genes *trxB* and *trxC* also exhibited upregulated expression (**Figure 3E**).

Under the oxidative stress induced by IBA or DS, respectively, the changes in gene expression related to the bacterial defense mechanism SOS response were analyzed. Among the five genes associated with the SOS response, *phoP* expression was increased 2.52- and 1.83-fold by IBA and DS (*p* < 0.05), respectively. *umuC* is a key component of the SOS response in *E. coli*, and its expression increased 2.52- and 2.27-fold (*p* < 0.05) after treatment with IBA and DS, respectively. In the DS-treated group, *recF* and *yebG* were upregulated by 1.39- and 1.58-fold, respectively.

Both IBA and DS clearly increased ROS generation in the conjugation system, leading to the upregulation of ROS- and SOS-related gene transcription. We proposed that both IBA and DS induced ROS overproduction in these strains, eventually contributing to the increased plasmid transfer ratio.

### 3.3 Both IBA and DS increased cell membrane permeability

**Figure 4:**
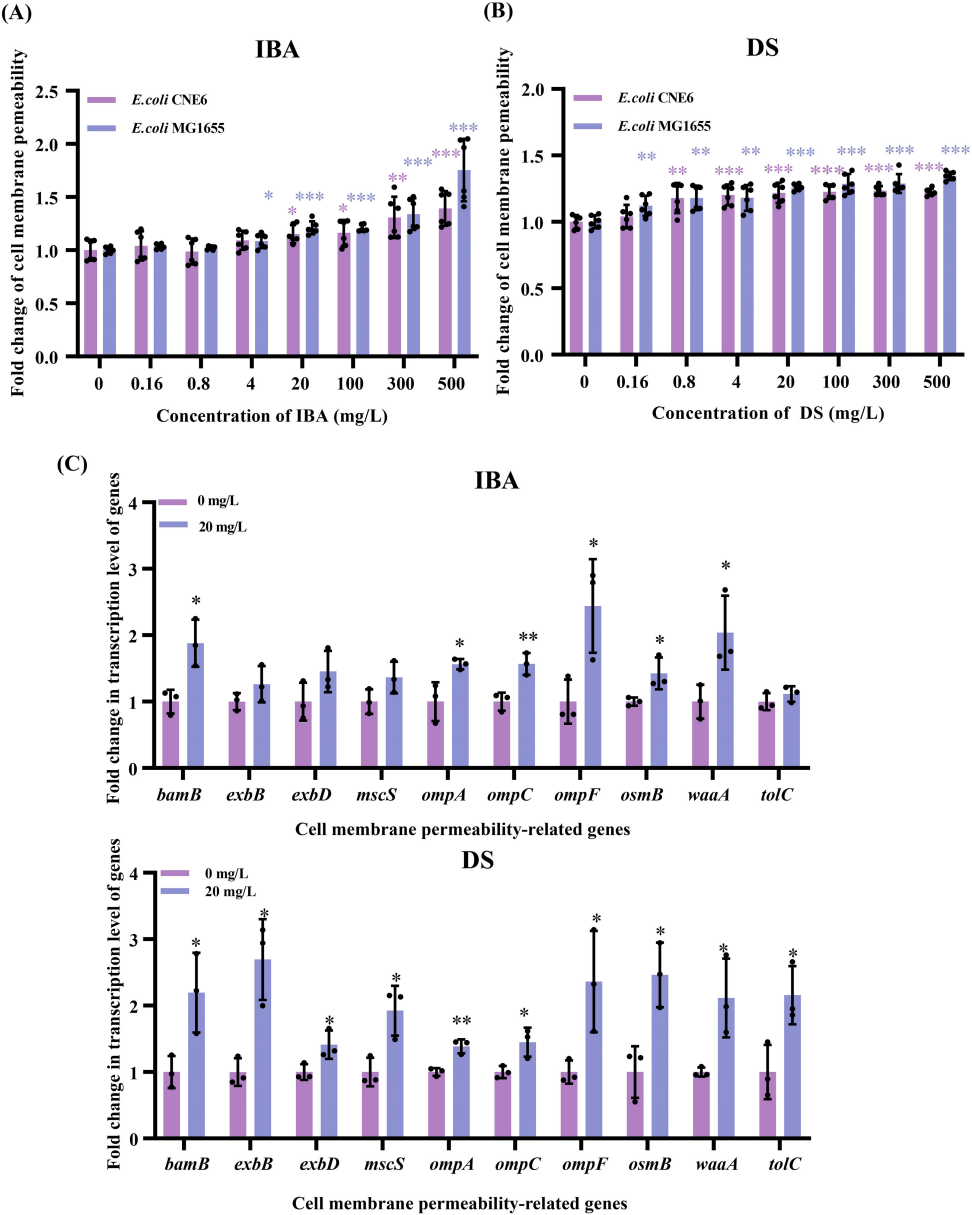
Changes related to the cell membrane permeability after exposure to IBA and DS. Fold changes in the cell membrane permeability by *E*. *coli* MG1655 and *E*. *coli* CNE6 after 2 h treatment of IBA **(A)** and DS **(B)** were shown. The results represent the mean ± SD of six biological samples. Fold changes in the transcription levels of cell membrane-related genes **(C)** in the conjugation systems were shown. The results represent the mean ± SD of three biological samples. Significant differences between the IBA or DS treatment groups at the different concentrations and the control group were tested with *t*-test and indicated by *p < 0.05, **p<0.01, and ***p< 0.001.

Bacterial cell membranes serve as a critical barrier impeding the horizontal transfer of ARGs. Conversely, an increased membrane permeability may promote the entry and exit of resistance plasmids. We used the PI staining method to evaluate the changes in the membrane permeability of the donor and recipient bacteria after IBA/DS treatment. After treatment with IBA or DS for 2 h, the membrane permeability of both the donor and recipient cells increased in a dose-dependent effect manner. At 4–500 mg/L, the membrane permeability of the donor bacteria increased by 1.09–1.75-fold (IBA) and 1.18–1.35-fold (DS), respectively. Meanwhile, the recipient bacteria showed a smaller increase, with 1.09–1.39-fold (IBA) and 1.20–1.23-fold (DS) increases in permeability (*p* < 0.05, **Figures 4A, B**).

The 20 mg/L IBA or DS treatment led to upregulation of the genes related to cell membrane permeability, respectively (**Figure 4C**). Among the ten genes associated with cell membrane permeability, the *omp* gene family exhibited upregulation, with expression of *ompA*, *ompC* and *ompF* increasing by 1.56–2.44-fold (IBA) and 1.39–2.36-fold (DS) (*p* < 0.05). In addition, *bamB* and *waaA* exhibited significant upregulation, with increases of 1.88–2.04-fold (IBA) and 2.11–2.20-fold (DS) (*p* < 0.05), respectively. After the DS treatment, *exbB* and *exbD* expression increased by 2.69- and 1.41-fold (*p* < 0.05) and genes encoding a transport channel (*tolC*), osmotic pressure regulator (*mscS*), and osmotically inducible lipoprotein (*osmB*) were all upregulated.

These results confirmed that both IBA and DS increased the cell membrane permeability of both donor and recipient cells and increased the transcription levels of cell membrane-related genes.

### 3.4 Both IBA and DS increased the plasmid copy number

**Figure 5:**
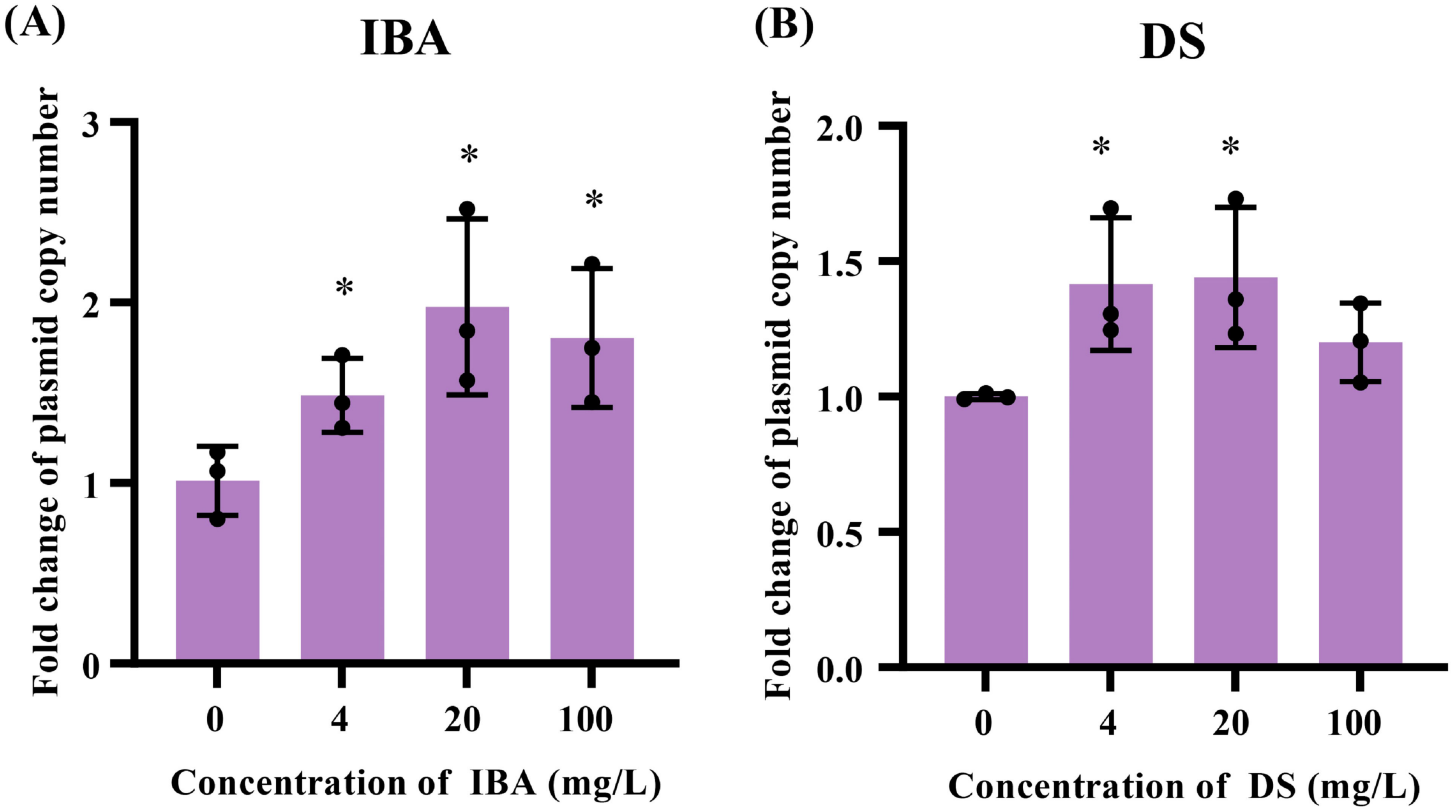
Changes related to the plasmid copies after exposure to IBA and DS. Fold changes in the plasmid copy numbers in the conjugation systems after 18 h treatment with IBA **(A)** and DS **(B)** were shown. The results represent the mean ± SD of three biological samples. Significant differences between the IBA or DS treatment groups at the different concentrations and the control group were tested with *t*-test and indicated by *p < 0.05.

Increasing the plasmid copy number in the host bacteria enhances the ratio of plasmid horizontal transfer. When the IBA concentration was 4–100 mg/L, the copy number increased by 1.47-, 1.95-, and 1.58-fold over that of the control (*p* < 0.05, **Figure 5A**; **Supplementary Table S9**). The plasmid copy numbers were 1.42-, 1.44-fold over the control group copy numbers after exposure to DS at concentrations of 4 and 20 mg/L, respectively (*p* < 0.05, **Figure 5B**).

### 3.5 Transcription levels of genes related to pilus generation, ATP synthesis, and the type IV secretion system were upregulated

**Figure 6:**
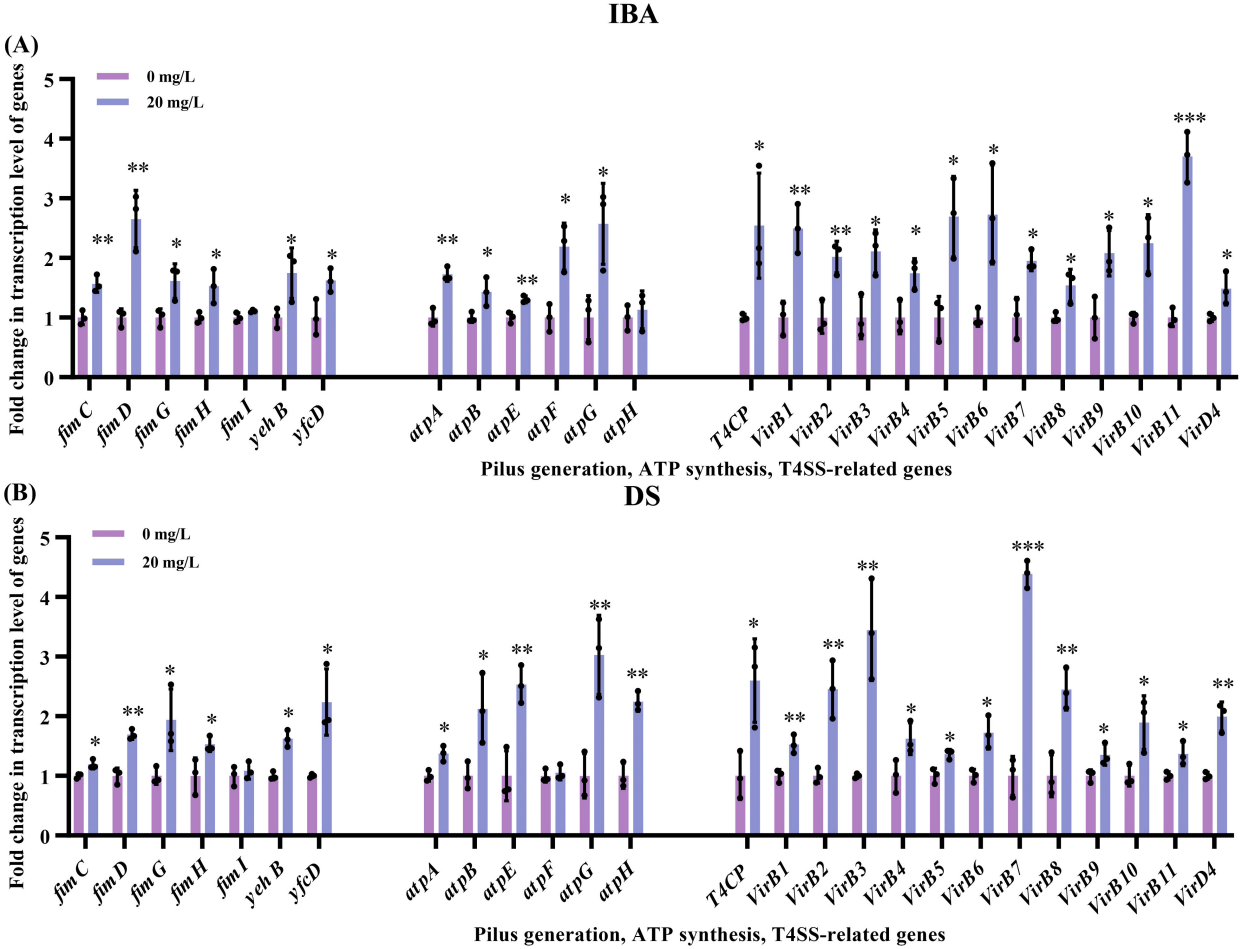
Changes related to the transcription levels of genes associated with pilus generation, ATP synthesis, and T4SS after exposure to IBA and DS. Fold changes in the transcription levels of genes related to pilus generation and ATP synthesis in the conjugation systems, as well as the T4SS of donor after 2 h treatment with IBA **(A)** and DS **(B)** were shown. The results represent the mean ± SD of three biological samples. Significant differences between the IBA or DS treatment groups and the control group were tested with *t*-test and indicated by *p < 0.05, **p<0.01, and ***p< 0.001.

In addition to the mechanisms previously discussed, the pilus generation, ATP synthesis, and T4SS pathways also regulate plasmid transfer. After exposure to 20 mg/L of IBA or DS, respectively, we observed upregulated transcription levels of genes related to pilus generation, ATP synthesis, and the T4SS (**Figure 6**). Among the seven genes involved in pilus generation, the transcription levels of *fimC*, *fimD, fimG, fimH* genes increased by 1.53–2.65-fold (IBA) and 1.19–1.94-fold (DS) over that of the control (*p* < 0.05, **Figure 6**, **Supplementary Table S9**). The transcription levels of *yehB* and *yfcD* increased by 1.62–1.75-fold (IBA) and 1.62–2.24-fold (DS) over that of the control group levels (*p* < 0.05, **Figure 6**; **Supplementary Table S9**). The genes encoding ATP may play a role in plasmid conjugative transfer by influencing cellular energy metabolism. Among the six ATP encoding genes (*atpA*, *atpB* and *atpE*–*H*), after treatment with IBA, the transcription levels of *atpA*, *atpB* and *atpE*–*G* increased by 1.31–2.57-fold (*p* < 0.05, **Figure 6A**). After treatment with DS, the transcription levels of *atpA*, *atpB*, *atpE*, *atpG* and *atpH* increased by 1.37–3.03-fold (*p* < 0.05, **Figure 6B**). The T4SS is a transmembrane channel structure that mediates the transfer of DNA between bacteria. IncI2 plasmids carry the *virB*/*D4* gene cluster for the synthesis of the T4SS. After exposure to IBA or DS, respectively, the transcription levels of 12 constituent element genes (*virB1–11* and *D4*) and the transport-coupled protein-coding genes *T4CP* increased by 1.48–3.70-fold (IBA) and 1.35–4.39-fold (DS) (*p* < 0.05, **Figure 6**; **Supplementary Table S9**).

## 4 Discussion

**Figure 7:**
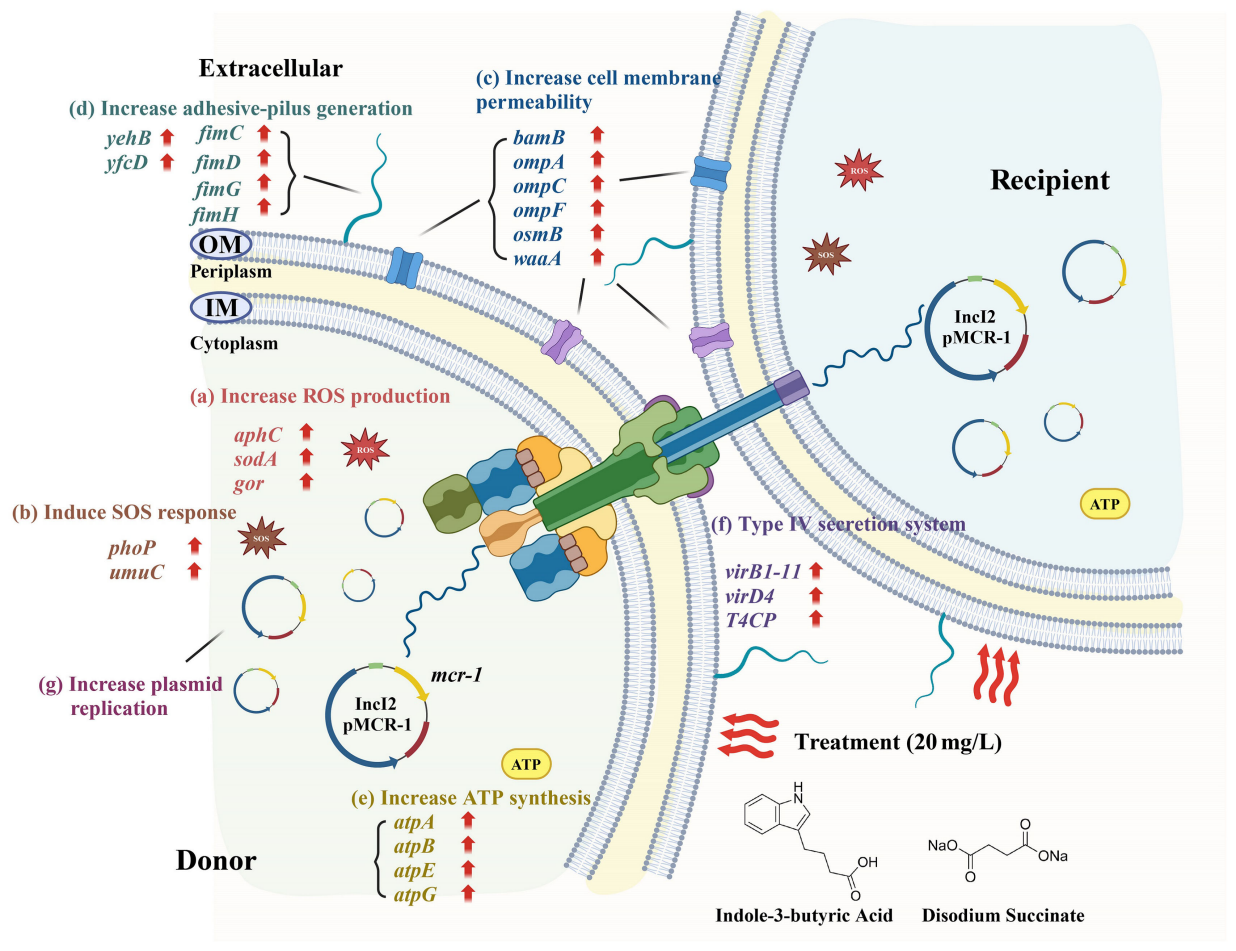
The metabolic pathway changes associated with the accelerated conjugative transfer of ARGs mediated by the IncI2 pMCR-1 under the influence of IBA and DS. The schematic diagram was created with BioRender.com.

The positive effects of gut microbiota metabolites on human health are gradually being recognized and accepted by the public (Krishnamurthy et al., 2023). For example, they can serve as medicinal ingredients in medical research (Lavelle and Sokol, 2020) and as ingredients in the food industry (Krautkramer et al., 2021). Because of their beneficial functions, their potential health risks are easily overlooked. This study primarily focuses on the impact of metabolites on the dissemination of antibiotic resistance plasmids among gut bacteria. *E. coli*, a major component of the gut microbiota and a carrier of multidrug resistance plasmids, served as a representative model for the transfer of plasmids. Using an established conjugation model, our study demonstrated that low concentrations of IBA or DS significantly promoted the horizontal transfer of ARGs through plasmid-mediated conjugation by increasing the production of ROS, the SOS response, cell membrane permeability, plasmid copy number, and enhancing the pilus generation, ATP synthesis, and the T4SS (**Figure 7**). Exposure to 20 mg/L of IBA or DS resulted in the highest level of plasmid transfer, respectively, after which the transfer ratio progressively declined with further increases in concentration. This phenomenon was similarly reported in the study in which indole-3-acetic acid (IAA) promoted plasmid transfer. IBA is the precursor of IAA and can be converted into IAA during the β-oxidation process in peroxisomes (Damodaran and Strader, 2019). Exposure to 50 mg/L of IAA significantly promoted plasmid conjugative transfer ratio, while inhibitory effects on conjugation gradually emerged at concentrations exceeding 100 mg/L (Zhao et al., 2023). The growth curves generated in this study indicated that high concentrations of IBA inhibited the growth of the donor bacteria. Therefore, the decrease in conjugative transfer ratio may be due to the bacteriostatic effect of IBA.

This study showed the correlation between the ROS levels and the plasmid transfer, which was confirmed by the addition of a ROS scavenger. After IBA treatment, both the donor and recipient bacteria exhibited increasing and then decreasing ROS levels as the IBA concentration increased. Donor bacteria ROS levels sharply decreased when the IBA concentration was 300–500 mg/L, probably due to high IBA concentration inhibiting the donor growth. In contrast, the ROS levels of the donor and recipient gradually increased as the concentration of DS increased, however, the conjugative transfer ratio decreased when the DS concentration exceeded 100 mg/L. It should be noted that excessively high ROS generation may irreversibly damage cell functions and inactivate the cells, thereby leading to a decreased conjugative transfer ratio (Zhang et al., 2019). An increase in the expression of ROS-related genes was also detected. In this study, the ROS-related genes encoding alkyl hydroperoxide reductase (*ahpC*), glutathione oxidoreductase (*gor*), and superoxide dismutase (*sodA*) were observed to be upregulated. It is previously known that oxidative stress caused by ROS activates the SOS response. Plasmid transfer promotion by the SOS response has been previously documented (Wang, et al., 2022). This study discovered that both IBA and DS treatment increased the transcription levels of SOS-related genes, specifically the DNA-binding transcription regulator genes *phoP* and the DNA repair gene *umuC*. Thus, the production of ROS and the SOS response may contribute to an increased conjugative transfer ratio.

The cell membrane usually acts as a barrier against the horizontal transfer of resistance plasmids (Yang et al., 2024). Increased donor cell membrane permeability significantly promoted resistance plasmids transfer (Yu et al., 2021). In this study, the cell membrane permeability increased continuously as IBA or DS concentrations increased. Specifically, after treatment with IBA or DS, the permeability of the donor cells increased more than that of the recipient cells. An increase in the transcription levels of cell membrane permeability-related genes was also found. The *omp* gene family is responsible for encoding outer membrane proteins (Mikheyeva et al., 2023). *bamB*, a key factor in the assembly of outer membrane proteins, is responsible for maintaining the integrity of the outer membrane (Knowles et al., 2009). *waaA* is responsible for the synthesis of lipopolysaccharides, thereby maintaining the permeability of the cell membrane (Mamat et al., 2009). The upregulation of these genes at the transcription levels led to an increase in membrane pore proteins, enhancing cell membrane permeability, thereby resulting in an increase in the transfer of resistance plasmids. All these findings support the view that the permeability of the cell membrane, particularly that of the donor cells, contributed to the conjugative transfer of the plasmids.

Additionally, several previous studies have confirmed that the upregulation of genes at the transcription levels involved in pilus generation, ATP synthesis, and the T4SS can promote plasmid transfer (Zhao et al., 2021; Zhu et al., 2024; He et al., 2022). This study found that after exposure to IBA or DS, respectively, the transcription levels of some genes related to pilus assembly (*fimC*, *fimD*) and adhesion (*fimG*, *fimH, yehB* and *yfcD*) were upregulated, which may possibly facilitate cell contact between the donor and recipient, and thus enhance the plasmid DNA transfer. ATP is a crucial energy source for cellular activities and facilitates transfer of resistant plasmid (Chen et al., 2005; Yu et al., 2020). The proteins encoded by *atpA*–*H* genes collectively form the ATP synthase, which catalyzes the synthesis of ATP (Prieto-alamo et al., 2000). We observed that after treatment with IBA or DS, respectively, the transcription levels of ATP-related genes were upregulated. Treatment with IBA or DS respectively significantly increased the plasmid copy number of the conjugation systems, which also implied an increase in the plasmid copy number carrying transfer-associated elements, such as the T4SS. The T4SS is a highly diverse super-family. The T4SS in IncI2 is encoded by the *virB/D4* gene cluster, which includes 12 core subunits and *T4CP*. Upon treatment with IBA or DS, respectively, the transcription levels of the T4SS component genes (*virB/D4*) of the donor bacteria increased. The upregulated transcription of this essential conjugation component may promote the self-transfer of the plasmid.

The concentrations of IBA and DS in the *in vivo* environment could be affect by a variety of factors, such as the complex intestinal environment, food intake, and the bacterial colonization sites. Therefore, the actual concentrations of IBA and DS in the gut have not yet been clearly determined. This study only revealed the potential risks of low-concentration IBA and DS in the quantitative spread of bacterial resistance *in vitro*. The further research is still needed to investigate the effects *in vivo.*

## 5 Conclusions

In this study, using a dual-fluorescence reporter system, intestinal metabolites IBA and DS that promote the transfer of IncI2 pMCR-1 at low concentrations were identified, and the molecular mechanisms underlying this phenomenon were explored. The effects and mechanisms of IBA or DS on ARGs horizontal transfer via plasmid-mediated conjugation were systematically investigated. The results showed that 4–100 mg/L IBA and DS all significantly promoted the transfer ratios of the plasmid by increasing the production of ROS, the SOS response, cell membrane permeability, plasmid copy number. The transcription of genes of the related pathways and of pilus, ATP, and the T4SS was upregulated. This research highlights the need for increased attention to the role of gut microbiota metabolites in promoting plasmid transfer, especially those considered beneficial to health and used in the food industry.

## Data Availability

All data needed to evaluate the conclusions in the paper are present in the paper and/or the Supplementary Materials “ Data Sheet”. For additional details of this study, please contact the corresponding authors LZ (lizhe@icdc.cn) and XL (luxin@icdc.cn).
