# Supplementary material for: Intestinal flora metabolites indole-3-butyric acid and disodium succinate promote IncI2 mcr-1-carrying plasmid transfer
Source: Front Cell Infect Microbiol. 2025 Jun 3;15:1564810. doi: 10.3389/fcimb.2025.1564810 (PMC12170664; doi:10.3389/fcimb.2025.1564810)
Supplement: Supplementary file 2 [file Image1.pdf]

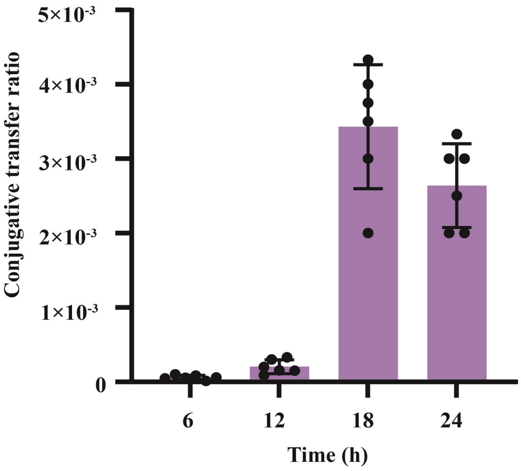

Supplementary Figure S1. The effect of conjugation time on the transfer ratio of IncI2 pMCR-1 in *E. coli* conjugation pairs. The plasmid transfer ratio at 6 h, 12 h, 18 h, and 24 h were shown. The results represent the mean ± SD of six biological samples.
