# Supplementary material for: Intestinal flora metabolites indole-3-butyric acid and disodium succinate promote IncI2 mcr-1-carrying plasmid transfer
Source: Front Cell Infect Microbiol. 2025 Jun 3;15:1564810. doi: 10.3389/fcimb.2025.1564810 (PMC12170664; doi:10.3389/fcimb.2025.1564810)
Supplement: Supplementary file 3 [file Image2.pdf]

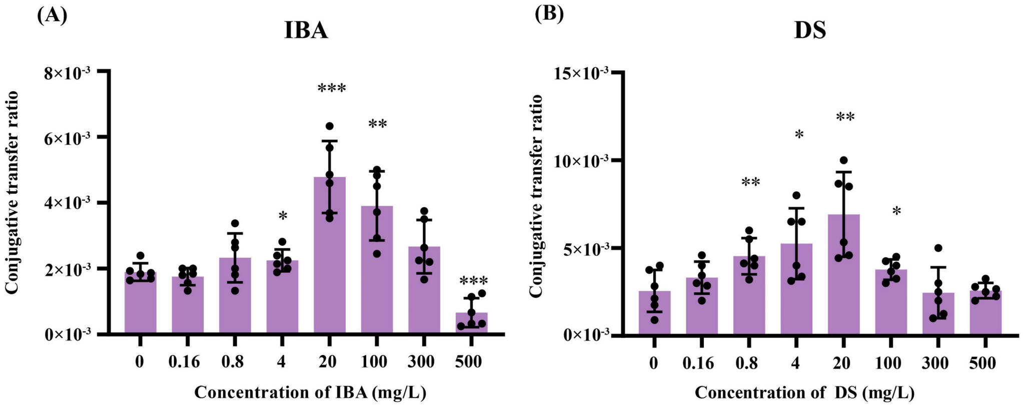

Supplementary Figure S2. The effects of IBA and DS on the transfer ratio of IncI2 pMCR-1 in *E. coli* conjugation pairs. The plasmid transfer ratio after 18 h of IBA (A) and DS (B) treatment were shown. The results represent the mean ± SD of six biological samples. Significant differences between the IBA or DS treatment groups at the different concentrations and the control group were tested by *t*-test and indicated by * $p < 0.05$, ** $p < 0.01$, and *** $p < 0.001$.
