# Supplementary material for: Intestinal flora metabolites indole-3-butyric acid and disodium succinate promote IncI2 mcr-1-carrying plasmid transfer
Source: Front Cell Infect Microbiol. 2025 Jun 3;15:1564810. doi: 10.3389/fcimb.2025.1564810 (PMC12170664; doi:10.3389/fcimb.2025.1564810)
Supplement: Supplementary file 4 [file Image3.pdf]

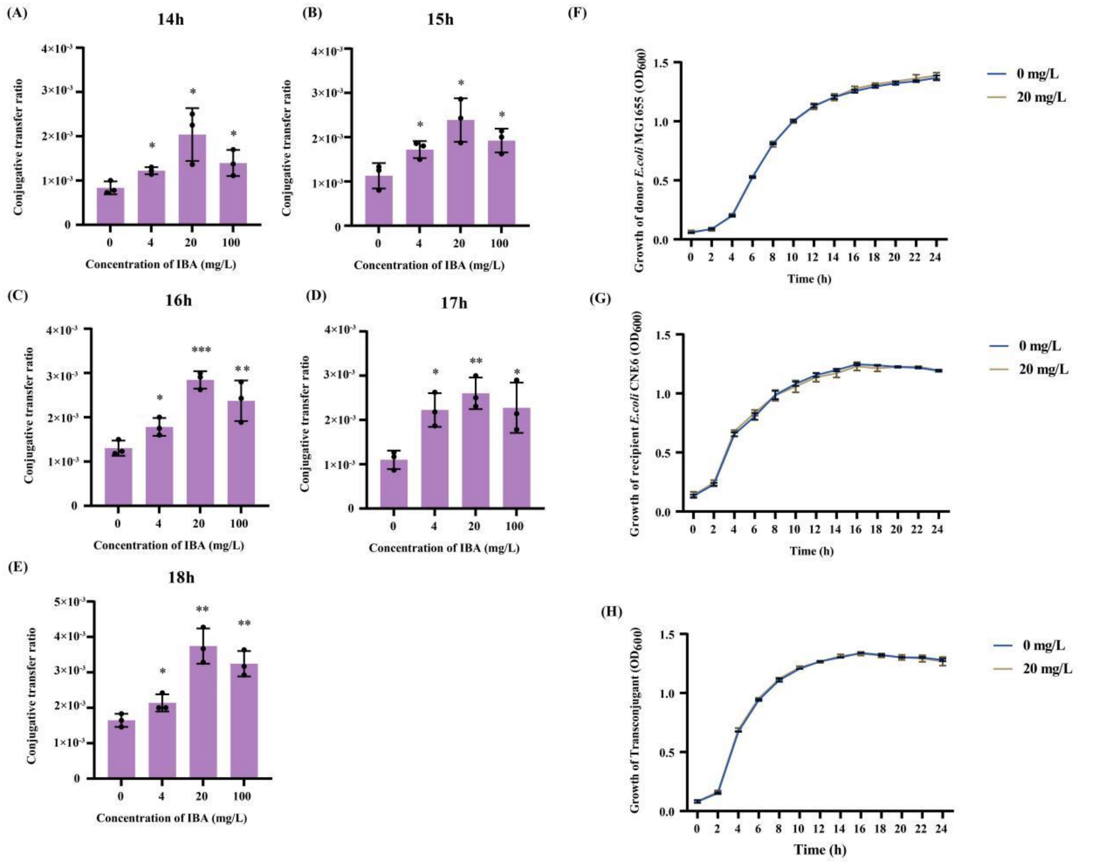

Supplementary Figure S3. The effect of various IBA concentrations treatments on the conjugative transfer ratio of the IncI2 pMCR-1 under different conjugation times and 20 mg/L IBA on the growth of donor, recipient, and transconjugant. The plasmid transfer ratios after conjugation for 14 h (A) , 15 h (B), 16 h (C), 17 h (D) and 18 h (E) under various IBA concentrations treatment were shown. The growth states of the donor (F), recipient (G), and transconjugant (H) of 20 mg/L IBA treatment and without IBA treatment were shown. The results represent the mean ± SD of three biological samples. Significant differences between the IBA treatment groups at the different concentrations and the control group were tested by *t*-test and indicated by * $p < 0.05$, ** $p < 0.01$, and *** $p < 0.001$.
