# Supplementary material for: Intestinal flora metabolites indole-3-butyric acid and disodium succinate promote IncI2 mcr-1-carrying plasmid transfer
Source: Front Cell Infect Microbiol. 2025 Jun 3;15:1564810. doi: 10.3389/fcimb.2025.1564810 (PMC12170664; doi:10.3389/fcimb.2025.1564810)
Supplement: Supplementary file 5 [file Image4.pdf]

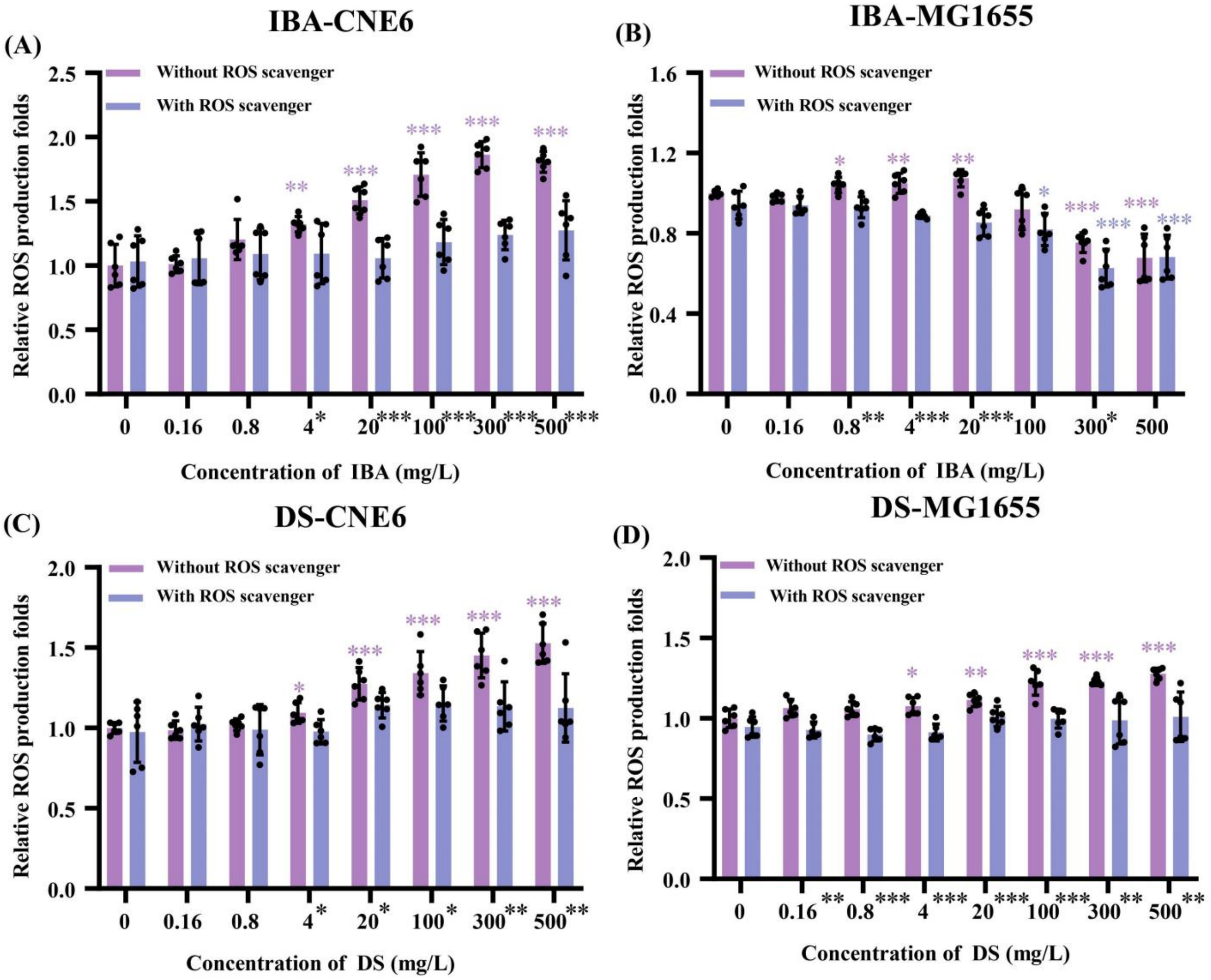

Supplementary Figure S4. Changes related to ROS with or without the ROS scavenger. Fold changes in ROS production by *E. coli* CNE6 and *E. coli* MG1655 under IBA (A, B) and DS (C, D) exposure for 2 h with or without the ROS scavenger were shown. The * on the numbers indicated inter-group differences, while the * in the graph represented intra-group differences. The results represent the mean ± SD of six biological samples. Significant differences between the IBA or DS treatment groups at the different concentrations and the control group were tested with *t*-test and indicated by * $p < 0.05$, ** $p < 0.01$, and *** $p < 0.001$.
