# Supplementary material for: Intestinal flora metabolites indole-3-butyric acid and disodium succinate promote IncI2 mcr-1-carrying plasmid transfer
Source: Front Cell Infect Microbiol. 2025 Jun 3;15:1564810. doi: 10.3389/fcimb.2025.1564810 (PMC12170664; doi:10.3389/fcimb.2025.1564810)
Supplement: Supplementary file 6 [file Table1.docx]

**Supplementary Table S1.** The information of bacterial strain and plasmids

|  | Genotype or phenotype | sources |
| --- | --- | --- |
| Strains (*Escherichia coli*) |  |  |
| CNE6 | No plasmid; Sm^R^ | This study |
| MG1655 | No plasmid; Rfp^R^ | Lab stock  (Blattner et al., 1997) |
| MG1655::*mCherry* | MG1655 containing the *mCherry* gene marker | This study |
| J53-pSH13G841 | J53 containing the pSH13G841 plasmid | Lab stock  (Yi et al., 2012) |
| SM10 λpir | *Thi thr leu tonA lacY supE recA*::*RP4-2-Tc*::*Mu* Km λpir | Lab stock (Simon et al., 1984) |
| J53-pSH13G841-*gfp* | J53 containing the pSH13G841-*gfp* plasmid | This study |
| MG1655::*mCherry*/pSH13G841*-gfp* | MG1655 containing the *mCherry* gene and pSH13G841-*gfp* plasmid | This study |
| Plasmid |  |  |
| pWM91 | Suicide plasmid; Amp^R^ | Lab stock (Metcalf et al., 1996) |
| pWM91-*mCherry* | pWM 91 containing the *mCherry* gene marker | This study |
| pUC18-*mCherry* | With the *mCherry* gene; Amp^R^ | Lab stock (Staal et al., 2019) |
| pSH13G841 | IncI2; PB^R^ | Lab stock (Lu et al., 2019) |
| pWM91-Cm | pWM91 containing the chloramphenicol resistance gene | Lab stock |
| pWM91-Cm-*gfp* | pWM 91-Cm containing the *gfp* gene marker | This study |
| pSH13G841-*gfp* | pSH13G841 containing the *gfp* gene marker | This study |
| pXG10-*gfp* | With the *gfp* gene | Lab stock (Urban and Vogel, 2007) |

**References:**

Blattenr, F. R., Plunkett, G. R., Bloch, C. A., Perna, N. T., Burland, V., Riley, M., et al. (1997), "The complete genome sequence of *Escherichia coli* K-12", *Science,* Vol. 277 No. 5331, pp. 1453-62. doi: 10.1126/science.277.5331.1453.

Lu, X., Zeng, M., Xu, J., Zhou, H., Gu, B. & Li, Z., et al. (2019), "Epidemiologic and genomic insights on *mcr-1*-harbouring Salmonella from diarrhoeal outpatients in Shanghai, China, 2006-2016", *EBioMedicine,* Vol. 42133-144. doi: 10.1016/j.ebiom.2019.03.006.

Metcalf, W. W., Jiang, W., Daniels, L. L., Kim, S. K., Haldimann, A. & Wanner, B. L. (1996), "Conditionally replicative and conjugative plasmids carrying lacZ alpha for cloning, mutagenesis, and allele replacement in bacteria", *Plasmid,* Vol. 35 No. 1, pp. 1-13. doi:10.1006/plas.1996.0001.

Simon, R., U. Priefer, and A. Piihler. (1983), "A broad host rangemobilization system for in vivo genetic engineering: transposonmutagenesis in gram negative bacteria", *Nature Biotechnology*, Vol. 1 No. 9, pp. 784–91. doi: 10.1038/nbt1183-784.

Staal, J., Alci, K., De Schamphelaire, W., Vanhoucke, M. & Beyaert, R. (2019), "Engineering a minimal cloning vector from a pUC18 plasmid backbone with an extended multiple cloning site", *Biotechniques,* Vol. 66 ,No. 6, pp. 254-259. doi: 10.2144/btn-2019-0014.

Urban, J. H. & Vogel, J. (2007), "Translational control and target recognition by *Escherichia coli* small RNAs in vivo", *Nucleic Acids Res,* Vol. 35 No. 3, pp. 1018-37. doi: 10.1093/nar/gkl1040.

Yi H, Cho YJ, Yong D, Chun J. (2012), "Genome sequence of *Escherichia coli* J53, a reference strain for genetic studies", *J Bacteriol,* Vol. 194 No. 14, pp. 3742-3. doi: 10.1128/JB.00641-12.
