# Supplementary material for: Intestinal flora metabolites indole-3-butyric acid and disodium succinate promote IncI2 mcr-1-carrying plasmid transfer
Source: Front Cell Infect Microbiol. 2025 Jun 3;15:1564810. doi: 10.3389/fcimb.2025.1564810 (PMC12170664; doi:10.3389/fcimb.2025.1564810)
Supplement: Supplementary file 7 [file Table2.docx]

**Supplementary Table S2.** Primers constructed for the dual-fluorescence reporter system

| Primer name | Primer sequences (5 ′ -3 ′) | products |
| --- | --- | --- |
| R-M91-XhoⅠ-F | GAATTGGGTACCGGGCCCCCCCTCGAGgatgatccaaccgcgtaaatctgac | *mCherry*-upstream homology arm |
| R-M91-up-R | CCGCTAGGTACTGTCAGATCGATCaataaattgcagcgttctgtaggc |  |
| *mCherr*y- promoter -F | gcctacagaacgctgcaatttattGATCGATCTGACAGTACCTAGCGG | *mCherry-*promoter |
| *mCherry*- promoter -R | GATAACGTCTTCGCTACTCGCCATGGTACCTTTCTCCTCTTTAATGAATTC |  |
| up-*mCherry*-F | GAATTCATTAAAGAGGAGAAAGGTACCATGGCGAGTAGCGAAGACGTTATC | *mCherry* |
| dn-*mCherry*-R | cggcctacatgatcgtgcaaattcGATCTATAAACGCAGAAAGGCCCAC |  |
| R-M91-dn-F | GTGGGCCTTTCTGCGTTTATAGATCgaatttgcacgatcatgtaggccg | *mCherry*-downstream homology arm |
| R-M91-BamHⅠ-R | GGCGGCCGCTCTAGAACTAGTGGATCCcgctcggtgattgttgagcacaac |  |
| M13-F | TGTAAAACGACGGCCAGT | Identification of the pWM 91-*mCherry* |
| M13-R | CAGGAAACAGCTATGACC |  |
| MG1655-F | cgttcgcgtaccgactttcg | Identification of *mCherry*-sequence |
| MG1655-R | ccactggatcaaacgcgacg |  |
| G-M91-Cm-BamhⅠ-F | tcagtgaagtgcttcatgtGGATCCCAAGAACAGCCATTGCAATTTGTGC | *gfp*-upstream homology arm |
| G-M91-Cm-up-R | ATGAGACGTTGATTGGCACGTAAGAGTGAAAGAACTGATAGCTGCTGGTTG |  |
| up-*gfp*-F | CAACCAGCAGCTATCAGTTCTTTCACTCTTACGTGCCAATCAACGTCTCAT | *gfp* |
| dn-*gfp*-R | CTGTCTATGTGTGACTGTTGAGCTGACAGGTCAGCAGATTTCATATGTCC |  |
| G-M91-Cm-dn-F | GGACATATGAAATCTGCTGACCTGTCAGCTCAACAGTCACACATAGACAG | *gfp*-downstream homology arm |
| G-M91-Cm-SacⅠ-R | CTAAAGGGAACAAAAGCTGGAGCTCTGCATGGAAAGCTGGCATTTATTCG |  |
| M91-Cm-*gfp*-F | gaggtgctccagtggcttctg | Identification of the pWM 91-Cm-*gfp* |
| M91-Cm-*gfp*-R | CAGGAAACAGCTATGACC |  |
| pSH13G841-F | CTGGAATTAAACGCAACCAG | *gfp*-sequence identification |
| pSH13G841-R | TGTCTTTACAGTGTGGTGCG |  |
