# Supplementary material for: Intestinal flora metabolites indole-3-butyric acid and disodium succinate promote IncI2 mcr-1-carrying plasmid transfer
Source: Front Cell Infect Microbiol. 2025 Jun 3;15:1564810. doi: 10.3389/fcimb.2025.1564810 (PMC12170664; doi:10.3389/fcimb.2025.1564810)
Supplement: Supplementary file 8 [file Table3.docx]

**Supplementary Table S3.** Primers targeting the conjugation-related gene

| Target genes | Primer sequences (5 ′-3 ′) |
| --- | --- |
| plasmid copies |  |
| *mcr-1* | TGGCGTTCAGTCATTAT |
|  | AGCTTACCCACCGAGTAGAT |
| ROS |  |
| *ahpC* | ACCAGGCATTCAAAAACGGC |
|  | GCCATGCTTTGTGGGTGAAG |
| *gor* | ATAACACGGGTGCAGTGGAG |
|  | TGATCGTCGCCATACTGCTC |
| *sodA* | CCAGAATTTGCCAACCTGCC |
|  | TCGATAGCCGCTTTCAGGTC |
| *trxB* | AATCAGCACAGGTTGCAGGT |
|  | GATGCTGCCGATTGCACAAA |
| *trxC* | CTGCGGTCACGACTTGTTTG |
|  | GCACTTTACCGCTACGCTCT |
| SOS |  |
| *phoP* | AATCATCAGCACCGGCACTT |
|  | TCAGGTCGATGACGCAGAAG |
| *recF* | GAATGACGCGACCAATCTGC |
|  | CGTGCAGATTGAAGATGCGG |
| *umuC* | TTAACGCTGCTACGCGATCT |
|  | GCTCGCTTCATCTGCCATTG |
| *uvrA* | GGTGATTGTCCCCACCGTAG |
|  | CAAATCCTCGCTCGCTTTCG |
| *yebG* | GAAAGGGCTTCACGTTGCTC |
|  | ACGTAGTCATTCGTGAGGGC |
| cell membrane permeability |  |
| *bamB* | CGCTGCCAATGTAGACATGC |
|  | GTGGAGCACTTCCGTTGGTA |
| *exbB* | CAGATCCAGGTCACGGCTTT |
|  | CCACTAACCTGGCAGTCGTT |
| *exbD* | TGTCCGCTCGGAAGAAGATG |
|  | GCGACGGTAGATGTGAAGGT |
| *mscS* | GGTTAGACAGTGACCCCTGC |
|  | TATCGCGCGGATGATTTCCA |
| *ompA* | GACCCTGGTTGTAAGCGTCA |
|  | GCACCGGAAGTACAGACCAA |
| *ompC* | AGTTGCGTTGTAGGTCTGGG |
|  | CGTCGGCGGTTCTATCACTT |
| *ompF* | CGCTATCAGGGTAACGGGAG |
|  | TCTGACAACAAACTGGGCGT |
| Target genes | Primer sequences (5 ′-3 ′) |
| *asmB* | ACCGTAGAGCACCTCAATGC |
|  | CAGCCCACTTATCGCCATCT |
| *tolC* | AACTGGAAAGTGCCCATCGT |
|  | TGGTCGCATCCAACACATCA |
| *waaA* | TCATCCTGGTACCCCGTCAT |
|  | CAGTGAACCGCCAACAAAGG |
| pilus generation |  |
| *fimC* | ACGCTGATTAACCCGACACC |
|  | TCACCCGGCAGTCAATTCTT |
| *fimD* | ACCAGCGAAACACACAATGC |
|  | TGGCGGTAATTCTTGCCCAT |
| *fimG* | GCATGATGTTGCGCTTGAGT |
|  | TGACCTGTAACGGGAAGTGC |
| *fimH* | AATCAGCGCACTTCCCGTTA |
|  | TGACCAGGCATTTACCGACC |
| *fimI* | CGGTGGTGAGTGAACGTGTA |
|  | CCAGTTTGCTGGAGGACGAT |
| *yehB* | GTTGGGCAAATCCACGTTCC |
|  | AGCGACTATAAAGCGTCGGG |
| *yfcD* | GAGACGCAGCTGAACAATGC |
|  | GTGTAGTCCAGGCCGATGAG |
| ATP synthesis |  |
| *atpA* | TCCAGGATACGGCCAGTACA |
|  | GTGTTATCCGCATTCACGGC |
| *atpB* | ATTGTCCAGAAGGTGGCTGG |
|  | GGTTTTGGTGCTGGTGGTTC |
| *atpE* | TTACTACGCGACAGCGAACA |
|  | GATCGGTATCGGCATCCTCG |
| *atpF* | TCGCCTGCTCGATGATTACC |
|  | CCATCGCGTTTGTCCTGTTC |
| *atpG* | GGTCGGAACCTGAGACATGG |
|  | TCGTTCTTCAACTCCGTGGG |
| *atpH* | CGGCACGCAGGTGAATAAAC |
|  | GTAGAACGCTGGCAGGACAT |
| T4SS |  |
| *T4CP* | AAACGCCATGAACACCTTCC |
|  | AAGACGTTTGCCACGTTTCC |
| *VirB1* | GGCAGTCCTGACGGACAATTA |
|  | TTTCACGGATTGATGGCACG |
| *VirB2* | TCGACACCGCAACAAATACCA |
|  | CAATAGACAAGCGCCATTCCT |
| *VirB3* | ACCGTCCTGCGTTAATTGCT |
|  | ACGGATGACTCTTAGTGCGTT |
| Target genes | Primer sequences (5 ′-3 ′) |
| *VirB4* | GCGAATGCCTGTCGAAGAAC |
|  | AACAGGACCGCGAGTAAAGG |
| *VirB5* | AAGGGCTGAGAGATTAACCACG |
|  | CATTCTCTAGTTGCCGTTCAGC |
| *VirB6* | TGCTCAACTCTTACTGGCGG |
|  | GCTGATGCAGAACGACTTCC |
| *VirB7* | TCATAATTGCCTCCGCGCTA |
|  | GTGTTTAACTCAAACCATCCCCC |
| *VirB8* | GGTTTCGCAATAGCCTCCCT |
|  | CGGCCTGTTCCTGGATAGTC |
| *VirB9* | GCCTGGTCAGTATCGGTCAG |
|  | GCTGACGGTGGTTTCGGATA |
| *VirB10* | CAGTACGCAGACGGAAAGG |
|  | AGTTAACGAACACACGGGCT |
| *VirB11* | GCTGCATCCAAACCACATCC |
|  | GCCAGTCGTGAAAATGAGGC |
| *VirD4* | CCCCGACACGTTCTGGTAAA |
|  | CGGTGAGAGCGTATCTGTCC |
| reference gene |  |
| *recA* | GTCAACCAGTTCGCCGTAGA |
|  | CCGCGTGAAAGTGGTGAAGA |
