# Supplementary material for: Intestinal flora metabolites indole-3-butyric acid and disodium succinate promote IncI2 mcr-1-carrying plasmid transfer
Source: Front Cell Infect Microbiol. 2025 Jun 3;15:1564810. doi: 10.3389/fcimb.2025.1564810 (PMC12170664; doi:10.3389/fcimb.2025.1564810)
Supplement: Supplementary file 9 [file Table4.docx]

**Supplementary Table S4.** Fold changes of the conjugation transfer ratio of the IncI2 pMCR-1 by 172 intestinal flora substrates

| Serial number | Intestinal flora metabolites | Concentration | Fold change of plasmid transfer ratio |
| --- | --- | --- | --- |
| 1 | 5-Hydroxyindole | 0.1 mM | 1.305 |
| 2 | Rhamnose | 0.1 mM | 0.942 |
| 3 | D-(+)-Trehalose dihydrate | 0.1 mM | 0.878 |
| 4 | Indole-3-butyric acid | 0.1 mM | 2.552 |
| 5 | Disodium succinate | 0.1 mM | 2.813 |
| 6 | Tartaric acid (disodium dihydrate) | 0.1 mM | 0.918 |
| 7 | Urea | 0.1 mM | 0.519 |
| 8 | Deoxycholic acid sodium salt | 0.1 mM | 0.708 |
| 9 | 2'-Deoxyadenosine-5'-monophosphate | 0.1 mM | 1.653 |
| 10 | Melatonin | 0.1 mM | 0.763 |
| 11 | 3-Hydroxyphenylacetic acid | 0.1 mM | 1.834 |
| 12 | Gallic acid (hydrate) | 0.1 mM | 0.700 |
| 13 | D-Ribose(mixture of isomers) | 0.1 mM | 0.716 |
| 14 | cis,cis-Muconic acid | 0.1 mM | 1.495 |
| 15 | Trimethylamine N-oxide | 0.1 mM | 0.425 |
| 16 | Syringic acid | 0.1 mM | 1.170 |
| 17 | Gallic acid | 0.1 mM | 2.218 |
| 18 | Benzoic acid | 0.1 mM | 1.287 |
| 19 | L-Tryptophan | 0.1 mM | 1.061 |
| 20 | Salicylic acid | 0.1 mM | 0.701 |
| 21 | Allantoin | 0.1 mM | 1.859 |
| 22 | Indole | 0.1 mM | 0.794 |
| 23 | Paraxanthine | 0.1 mM | 0.980 |
| 24 | trans-Cinnamic acid | 0.1 mM | 1.303 |
| 25 | Vitamin B12 | 0.1 mM | 1.154 |
| 26 | L-Valine | 0.1 mM | 1.473 |
| 27 | p-Cresyl sulfate | 0.1 mM | 0.505 |
| 28 | 4-Hydroxyphenylpyruvic acid | 0.1 mM | 0.522 |
| 29 | Phenyl acetate | 0.1 mM | 1.001 |
| 30 | N-Methylnicotinamide | 0.1 mM | 0.719 |
| 31 | Pimelic acid | 0.1 mM | 0.512 |
| 32 | 2-Phenylpropionic acid | 0.1 mM | 1.112 |
| 33 | L-Tartaric acid | 0.1 mM | 0.937 |
| 34 | Nonadecanoic acid | 0.1 mM | 0.777 |
| 35 | (R)-3-Hydroxybutanoic acid | 0.1 mM | 0.745 |
| 36 | Thiamine nitrate | 0.1 mM | 0.908 |
| 37 | Glycolic acid | 0.1 mM | 0.649 |
| 38 | Imidazoleacetic acid (hydrochloride) | 0.1 mM | 1.246 |
| 39 | Rhamnose (monohydrate) | 0.1 mM | 2.066 |
| 40 | Isovaleric acid | 0.1 mM | 1.254 |
| 41 | alpha-D-glucose | 0.1 mM | 1.396 |
| Serial number | Intestinal flora metabolites | Concentration | Fold change of plasmid transfer ratio |
| 42 | Sodium Salicylate | 0.1 mM | 0.647 |
| 43 | Phenylacetylglycine | 0.1 mM | 0.585 |
| 44 | Vanillylmandelic acid | 0.1 mM | 1.256 |
| 45 | Succinic acid | 0.1 mM | 0.653 |
| 46 | Homovanillic acid | 0.1 mM | 1.616 |
| 47 | Norepinephrine (hydrochloride) | 0.1 mM | 0.501 |
| 48 | L-Leucyl-L-alanine | 0.1 mM | 1.217 |
| 49 | D-Arabitol | 0.1 mM | 1.565 |
| 50 | Taurochenodeoxycholic acid (sodium) | 0.1 mM | 2.148 |
| 51 | Salicyluric acid | 0.1 mM | 1.389 |
| 52 | Folic acid | 0.1 mM | 1.096 |
| 53 | Glycochenodeoxycholic acid | 0.1 mM | 1.154 |
| 54 | 4-Hydroxybenzoic acid | 0.1 mM | 0.545 |
| 55 | p-Cresyl sulfate (potassium) | 0.1 mM | 1.806 |
| 56 | Magnesium acetate tetrahydrate | 0.1 mM | 1.140 |
| 57 | Taurocholic acid | 0.1 mM | 0.778 |
| 58 | 4-Pyridoxic acid | 0.1 mM | 0.593 |
| 59 | 2'-Deoxyuridine | 0.1 mM | 0.696 |
| 60 | Glycoursodeoxycholic acid | 0.1 mM | 1.352 |
| 61 | Glycocholic acid | 0.1 mM | 1.270 |
| 62 | Formic acid (ammonium) | 0.1 mM | 1.433 |
| 63 | 4-Hydroxycyclohexanecarboxylic acid | 0.1 mM | 1.043 |
| 64 | (S)-Leucic acid | 0.1 mM | 1.095 |
| 65 | Dihydrocaffeic acid | 0.1 mM | 1.494 |
| 66 | Xanthosine | 0.1 mM | 0.700 |
| 67 | trans-3-Indoleacrylic acid | 0.1 mM | 0.539 |
| 68 | DL-Glutamine | 0.1 mM | 0.956 |
| 69 | 2,5-Dihydroxybenzoic acid | 0.1 mM | 1.136 |
| 70 | 3-Hydroxybenzoic acid | 0.1 mM | 1.502 |
| 71 | Emavusertib | 0.1 mM | 0.502 |
| 72 | DL-3-Phenyllactic acid | 0.1 mM | 0.623 |
| 73 | Taurocholic acid (sodium salt hydrate) | 0.1 mM | 1.638 |
| 74 | Pyridoxine (hydrochloride) | 0.1 mM | 0.886 |
| 75 | L-Dihydroorotic acid | 0.1 mM | 1.109 |
| 76 | L-Ascorbic acid | 0.1 mM | 1.243 |
| 77 | Quinolinic acid | 0.1 mM | 0.855 |
| 78 | 3-Hydroxyhippuric acid | 0.1 mM | 1.874 |
| 79 | 3-Indolepropionic acid | 0.1 mM | 0.813 |
| 80 | 2'-Deoxycytidine | 0.1 mM | 0.992 |
| 81 | Dihydrouracil | 0.1 mM | 1.074 |
| 82 | Glycodeoxycholic acid (monohydrate) | 0.1 mM | 1.328 |
| 83 | Biotin | 0.1 mM | 1.768 |
| 84 | Riboflavin | 0.1 mM | 0.661 |
| Serial number | Intestinal flora metabolites | Concentration | Fold change of plasmid transfer ratio |
| 85 | N-Acetylputrescine hydrochloride | 0.1 mM | 0.503 |
| 86 | N-Acetyl-D-glucosamine | 0.1 mM | 0.986 |
| 87 | Phenylpyruvic acid | 0.1 mM | 1.027 |
| 88 | 5-Hydroxymethyl-2-furancarboxylic acid | 0.1 mM | 0.553 |
| 89 | Methyl 2-(1H-indol-3-yl)acetate | 0.1 mM | 0.851 |
| 90 | 4-Aminobenzoic acid | 0.1 mM | 0.840 |
| 91 | 3,4-Dihydroxybenzeneacetic acid | 0.1 mM | 0.923 |
| 92 | Phloretin | 0.1 mM | 1.293 |
| 93 | Homogentisic acid | 0.1 mM | 0.535 |
| 94 | Uridine 5'-monophosphate | 0.1 mM | 0.609 |
| 95 | Protocatechuic acid | 0.1 mM | 0.942 |
| 96 | Glycochenodeoxycholic acid (sodium salt) | 0.1 mM | 0.517 |
| 97 | Skatole | 0.1 mM | 0.503 |
| 98 | Tyrosol | 0.1 mM | 2.159 |
| 99 | Pyrogallol | 0.1 mM | 0.685 |
| 100 | 3-Methyl-2-oxobutanoic acid | 0.1 mM | 1.229 |
| 101 | Hypoxanthine | 0.1 mM | 0.806 |
| 102 | Lithocholic acid | 0.1 mM | 1.101 |
| 103 | Stearic acid | 0.1 mM | 0.714 |
| 104 | Pyruvic acid | 0.1 mM | 0.844 |
| 105 | Phenylacetylglutamine | 0.1 mM | 1.210 |
| 106 | N-Acetyl-L-glutamic acid | 0.1 mM | 1.524 |
| 107 | 2-Hydroxyhexanoic acid | 0.1 mM | 0.669 |
| 108 | 5'-Methylthioadenosine | 0.1 mM | 0.820 |
| 109 | 2,3-Butanediol | 0.1 mM | 1.146 |
| 110 | Creatine | 0.1 mM | 1.902 |
| 111 | N-(5-Aminopentyl)acetamide | 0.1 mM | 1.726 |
| 112 | Inosinic acid (disodium)(hydrate)(1:2:X) | 0.1 mM | 1.096 |
| 113 | D-(+)-Trehalose | 0.1 mM | 1.095 |
| 114 | D-Alanine | 0.1 mM | 1.029 |
| 115 | L-Ornithine | 0.1 mM | 0.719 |
| 116 | Spermidine (hydrochloride) | 0.1 mM | 1.718 |
| 117 | L-Asparagine | 0.1 mM | 0.701 |
| 118 | 2'-Deoxycytidine-5'-monophosphoric acid | 0.1 mM | 1.376 |
| 119 | D-(-)-Lactic acid (sodium) | 0.1 mM | 1.514 |
| 120 | L-Arginine (hydrochloride) | 0.1 mM | 1.043 |
| 121 | 3-Amino-2-methylpropanoic acid | 0.1 mM | 0.736 |
| 122 | L-Gulose | 0.1 mM | 1.672 |
| 123 | L-Lysine hydrochloride | 0.1 mM | 1.557 |
| 124 | L-Histidine | 0.1 mM | 1.196 |
| 125 | Thiamine monochloride | 0.1 mM | 0.918 |
| 126 | Spermine (tetrahydrochloride) | 0.1 mM | 0.993 |
| 127 | D-Mannitol | 0.1 mM | 1.056 |
| Serial number | Intestinal flora metabolites | Concentration | Fold change of plasmid transfer ratio |
| 128 | L-Threonine | 0.1 mM | 0.997 |
| 129 | N-Methylsarcosine | 0.1 mM | 0.848 |
| 130 | Cytidine 5'-monophosphate | 0.1 mM | 1.435 |
| 131 | Glycine | 0.1 mM | 0.927 |
| 132 | L-Aspartic acid | 0.1 mM | 1.302 |
| 133 | Flavin adenine dinucleotide (disodium salt) | 0.1 mM | 0.557 |
| 134 | 2-Amino-5-ureidopentanoic acid | 0.1 mM | 1.103 |
| 135 | L-Serine | 0.1 mM | 0.694 |
| 136 | L-Lysine hydrate | 0.1 mM | 1.208 |
| 137 | L-Leucine | 0.1 mM | 0.600 |
| 138 | L-Glutamic acid | 0.1 mM | 0.956 |
| 139 | Inosinic acid | 0.1 mM | 1.316 |
| 140 | L-Lysine | 0.1 mM | 0.899 |
| 141 | D-Galacturonic acid (hydrate) | 0.1 mM | 0.829 |
| 142 | (S)-3,4-Dihydroxybutyric acid (lithium hydrate) | 0.1 mM | 1.056 |
| 143 | L-Ascorbic acid (sodium salt) | 0.1 mM | 1.066 |
| 144 | L-Phenylalanine | 0.1 mM | 1.347 |
| 145 | 5-Aminovaleric acid | 0.1 mM | 0.860 |
| 146 | 2,6-Diaminoheptanedioic acid | 0.1 mM | 1.058 |
| 147 | Spermine | 0.1 mM | 1.154 |
| 148 | Uridine 5′-diphosphoglucose (disodium salt) | 0.1 mM | 1.103 |
| 149 | N-Acetylornithine | 0.1 mM | 1.454 |
| 150 | L-Methionine | 0.1 mM | 1.354 |
| 151 | N-Acetylneuraminic acid | 0.1 mM | 0.981 |
| 152 | Aminomalonic acid | 0.1 mM | 1.001 |
| 153 | Hydrocinnamic acid | 0.1 mM | 1.134 |
| 154 | Pipecolic acid | 0.1 mM | 0.944 |
| 155 | Sodium 3-methyl-2-oxobutanoate | 0.1 mM | 1.688 |
| 156 | Dimethyl sulfone | 0.1 mM | 0.763 |
| 157 | Ribitol | 0.1 mM | 0.611 |
| 158 | γ-Aminobutyric acid | 0.1 mM | 1.306 |
| 159 | Phosphorylethanolamine | 0.1 mM | 0.824 |
| 160 | 2,5-Furandicarboxylic acid | 0.1 mM | 1.406 |
| 161 | (2-Aminoethyl)phosphonic acid | 0.1 mM | 1.254 |
| 162 | L-Proline | 0.1 mM | 1.402 |
| 163 | NSC 16590 | 0.1 mM | 1.066 |
| 164 | Cholesterol | 0.1 mM | 1.245 |
| 165 | Deoxycholic acid | 0.1 mM | 1.158 |
| 166 | 5-Hydroxyindole-3-acetic acid | 0.1 mM | 0.796 |
| 167 | Hydroxytyrosol | 0.1 mM | 1.829 |
| 168 | L-Tyrosine | 0.02 mM | 1.712 |
| 169 | Lecithin | 0.02 mM | 1.772 |
| 170 | Vitamin K | 30 mg/L | 1.538 |
| Serial number | Intestinal flora metabolites | Concentration | Fold change of plasmid transfer ratio |
| 171 | Cyclic AMP | 0.1 mM | 2.096 |
| 172 | L-Cysteine | 0.1 mM | 2.160 |
