# Supplementary material for: Intestinal flora metabolites indole-3-butyric acid and disodium succinate promote IncI2 mcr-1-carrying plasmid transfer
Source: Front Cell Infect Microbiol. 2025 Jun 3;15:1564810. doi: 10.3389/fcimb.2025.1564810 (PMC12170664; doi:10.3389/fcimb.2025.1564810)
Supplement: Supplementary file 10 [file Table5.docx]

**Supplementary Table S5.** The raw data of various IBA concentration treatments on the conjugation system, including the number of recipients, transconjugants, and the conjugative transfer ratio under different conjugation times.

| Conjugative time | Concentration of IBA | The number of transconjugants (10^3^) | | | The number of recipient (10^6^) | | | Conjugative transfer ratio (10^-3^) | | |
| --- | --- | --- | --- | --- | --- | --- | --- | --- | --- | --- |
| 14 h | Control group  （0 mg/L） | 10 | 7 | 8 | 10 | 9 | 11 | 1.000 | 0.778 | 0.727 |
|  | Treatment group  （4 mg/L） | 13 | 11 | 8 | 10 | 9 | 7 | 1.300 | 1.222 | 1.143 |
|  | Treatment group  （20 mg/L） | 15 | 18 | 10 | 11 | 8 | 4 | 1.364 | 2.250 | 2.500 |
|  | Treatment group  （100 mg/L） | 11 | 10 | 17 | 8 | 9 | 10 | 1.375 | 1.111 | 1.700 |
| 15 h | Control group  （0 mg/L） | 15 | 12 | 4 | 12 | 9 | 5 | 1.250 | 1.333 | 0.800 |
|  | Treatment group  （4 mg/L） | 13 | 18 | 10 | 7 | 10 | 7 | 1.857 | 1.800 | 1.429 |
|  | Treatment group  （20 mg/L） | 15 | 17 | 20 | 8 | 7 | 7 | 1.875 | 2.429 | 2.857 |
|  | Treatment group  （100 mg/L） | 15 | 20 | 13 | 7 | 10 | 8 | 2.143 | 2.000 | 1.625 |
| 16 h | Control group  （0 mg/L） | 13 | 15 | 16 | 11 | 10 | 13 | 1.182 | 1.500 | 1.231 |
|  | Treatment group  （4 mg/L） | 16 | 21 | 24 | 10 | 12 | 12 | 1.600 | 1.750 | 2.000 |
|  | Treatment group  （20 mg/L） | 36 | 21 | 32 | 12 | 8 | 11 | 3.000 | 2.625 | 2.909 |
|  | Treatment group  （100 mg/L） | 28 | 34 | 17 | 10 | 14 | 9 | 2.800 | 2.429 | 1.889 |
| 17 h | Control group  （0 mg/L） | 19 | 13 | 14 | 15 | 15 | 12 | 1.267 | 0.867 | 1.167 |
|  | Treatment group  （4 mg/L） | 28 | 24 | 21 | 15 | 11 | 8 | 1.867 | 2.182 | 2.625 |
|  | Treatment group  （20 mg/L） | 30 | 25 | 34 | 13 | 9 | 12 | 2.308 | 2.778 | 2.833 |
|  | Treatment group  （100 mg/L） | 25 | 30 | 29 | 14 | 14 | 10 | 1.786 | 2.143 | 2.900 |
| 18 h | Control group  （0 mg/L） | 23 | 22 | 19 | 14 | 12 | 13 | 1.643 | 1.833 | 1.462 |
|  | Treatment group  （4 mg/L） | 22 | 29 | 26 | 11 | 12 | 13 | 2.000 | 2.417 | 2.000 |
|  | Treatment group  （20 mg/L） | 33 | 47 | 46 | 9 | 11 | 14 | 3.667 | 4.273 | 3.286 |
|  | Treatment group  （100 mg/L） | 38 | 40 | 41 | 12 | 11 | 14 | 3.167 | 3.636 | 2.929 |

For each incubation time (14 h, 15 h, 16 h, 17 h, and 18 h) and each IBA concentration (0 mg/L, 4 mg/L, 20 mg/L and 100 mg/L), there are three biological replicates of raw data. We performed a total of sixty biological replicates experiments. The recipient bacteria and transconjugants in the each biological replicate experiment were counted, and the conjugative transfer ratio was calculated for each biological replicate experiment.
