# Supplementary material for: Intestinal flora metabolites indole-3-butyric acid and disodium succinate promote IncI2 mcr-1-carrying plasmid transfer
Source: Front Cell Infect Microbiol. 2025 Jun 3;15:1564810. doi: 10.3389/fcimb.2025.1564810 (PMC12170664; doi:10.3389/fcimb.2025.1564810)
Supplement: Supplementary file 11 [file Table6.docx]

**Supplementary Table S6.** The raw data on the growth states of recipient in the 20 mg/L IBA treatment group and control group.

| Time | Control group (0 mg/L) | | | Treatment group (20 mg/L) | | |
| --- | --- | --- | --- | --- | --- | --- |
| 0 h | 0.121 | 0.145 | 0.131 | 0.120 | 0.132 | 0.171 |
| 2 h | 0.215 | 0.243 | 0.230 | 0.221 | 0.240 | 0.267 |
| 4 h | 0.673 | 0.652 | 0.638 | 0.691 | 0.672 | 0.675 |
| 6 h | 0.833 | 0.809 | 0.776 | 0.863 | 0.826 | 0.819 |
| 8 h | 1.020 | 0.991 | 0.949 | 1.033 | 0.974 | 0.943 |
| 10 h | 1.102 | 1.082 | 1.063 | 1.115 | 1.049 | 1.015 |
| 12h | 1.171 | 1.158 | 1.138 | 1.178 | 1.128 | 1.104 |
| 14 h | 1.208 | 1.199 | 1.186 | 1.214 | 1.156 | 1.147 |
| 16 h | 1.257 | 1.247 | 1.238 | 1.269 | 1.217 | 1.203 |
| 18 h | 1.239 | 1.241 | 1.234 | 1.243 | 1.204 | 1.191 |
| 20 h | 1.221 | 1.230 | 1.222 | 1.227 | 1.231 | 1.218 |
| 22 h | 1.214 | 1.225 | 1.215 | 1.222 | 1.232 | 1.223 |
| 24 h | 1.186 | 1.201 | 1.187 | 1.192 | 1.202 | 1.198 |

For recipient strains, six biological replicates experiments were performed, with three biological replicates experiments for control group without IBA and three biological replicates experiments for 20 mg/L IBA treatment group. The OD_600_ values of each biological replicate experiment was measured every two hours at 37℃.
