# Supplementary material for: Intestinal flora metabolites indole-3-butyric acid and disodium succinate promote IncI2 mcr-1-carrying plasmid transfer
Source: Front Cell Infect Microbiol. 2025 Jun 3;15:1564810. doi: 10.3389/fcimb.2025.1564810 (PMC12170664; doi:10.3389/fcimb.2025.1564810)
Supplement: Supplementary file 12 [file Table7.docx]

**Supplementary Table S7.** The raw data on the growth states of donor in the 20 mg/L IBA treatment group and control group.

| Time | Control group (0 mg/L) | | | Treatment group (20 mg/L) | | |
| --- | --- | --- | --- | --- | --- | --- |
| 0 h | 0.056 | 0.060 | 0.060 | 0.078 | 0.057 | 0.056 |
| 2 h | 0.082 | 0.089 | 0.092 | 0.101 | 0.075 | 0.080 |
| 4 h | 0.190 | 0.203 | 0.204 | 0.210 | 0.193 | 0.214 |
| 6 h | 0.520 | 0.532 | 0.525 | 0.537 | 0.529 | 0.538 |
| 8 h | 0.807 | 0.805 | 0.818 | 0.832 | 0.801 | 0.787 |
| 10 h | 1.004 | 0.995 | 1.013 | 1.013 | 1.005 | 0.982 |
| 12h | 1.139 | 1.115 | 1.137 | 1.150 | 1.131 | 1.100 |
| 14 h | 1.215 | 1.188 | 1.209 | 1.225 | 1.214 | 1.173 |
| 16 h | 1.271 | 1.243 | 1.253 | 1.294 | 1.279 | 1.253 |
| 18 h | 1.306 | 1.283 | 1.295 | 1.324 | 1.308 | 1.306 |
| 20 h | 1.336 | 1.314 | 1.314 | 1.341 | 1.335 | 1.337 |
| 22 h | 1.349 | 1.335 | 1.336 | 1.400 | 1.341 | 1.349 |
| 24 h | 1.391 | 1.353 | 1.361 | 1.415 | 1.381 | 1.365 |

For donor strains, six biological replicates experiments were performed, with three biological replicates experiments for control group without IBA and three biological replicates experiments for 20 mg/L IBA treatment group. The OD_600_ values of each biological replicate experiment was measured every two hours at 37℃.
