# Supplementary material for: Intestinal flora metabolites indole-3-butyric acid and disodium succinate promote IncI2 mcr-1-carrying plasmid transfer
Source: Front Cell Infect Microbiol. 2025 Jun 3;15:1564810. doi: 10.3389/fcimb.2025.1564810 (PMC12170664; doi:10.3389/fcimb.2025.1564810)
Supplement: Supplementary file 13 [file Table8.docx]

**Supplementary Table S8.** The raw data on the growth states of transconjugant in the 20 mg/L IBA treatment group and control group.

| Time | Control group (0 mg/L) | | | Treatment group (20 mg/L) | | |
| --- | --- | --- | --- | --- | --- | --- |
| 0 h | 0.093 | 0.068 | 0.084 | 0.078 | 0.093 | 0.072 |
| 2 h | 0.170 | 0.147 | 0.171 | 0.154 | 0.158 | 0.144 |
| 4 h | 0.703 | 0.692 | 0.677 | 0.673 | 0.672 | 0.674 |
| 6 h | 0.952 | 0.959 | 0.954 | 0.935 | 0.950 | 0.937 |
| 8 h | 1.112 | 1.119 | 1.130 | 1.094 | 1.123 | 1.110 |
| 10 h | 1.210 | 1.213 | 1.228 | 1.203 | 1.206 | 1.207 |
| 12h | 1.263 | 1.257 | 1.275 | 1.266 | 1.272 | 1.268 |
| 14 h | 1.299 | 1.314 | 1.326 | 1.304 | 1.304 | 1.299 |
| 16 h | 1.319 | 1.332 | 1.349 | 1.339 | 1.341 | 1.339 |
| 18 h | 1.303 | 1.315 | 1.336 | 1.329 | 1.323 | 1.315 |
| 20 h | 1.282 | 1.297 | 1.325 | 1.300 | 1.306 | 1.298 |
| 22 h | 1.271 | 1.282 | 1.323 | 1.298 | 1.307 | 1.296 |
| 24 h | 1.240 | 1.255 | 1.310 | 1.272 | 1.291 | 1.275 |

For transconjugant strains, six biological replicates experiments were performed, with three biological replicates experiments for control group without IBA and three biological replicates experiments for 20 mg/L IBA treatment group. The OD_600_ values of each biological replicate experiment was measured every two hours at 37℃.
