# Supplementary material for: Intestinal flora metabolites indole-3-butyric acid and disodium succinate promote IncI2 mcr-1-carrying plasmid transfer
Source: Front Cell Infect Microbiol. 2025 Jun 3;15:1564810. doi: 10.3389/fcimb.2025.1564810 (PMC12170664; doi:10.3389/fcimb.2025.1564810)
Supplement: Supplementary file 14 [file Table9.docx]

**Supplementary Table S9.** The fold changes in gene transcription levels after 2 h of treatment with 20 mg/L IBA and DS.

| ROS | IBA | DS |
| --- | --- | --- |
| *aphC* | 1.187^*^ | 2.077^*^ |
| *sodA* | 1.827^*^ | 1.971^**^ |
| *trxB* | 1.228 | 1.666^*^ |
| *trxC* | 1.063 | 1.400^*^ |
| *gor* | 1.844^**^ | 2.249^*^ |
| SOS |  |  |
| *phoP* | 2.523^*^ | 1.827^*^ |
| *recF* | 1.110 | 1.392^*^ |
| *umuC* | 2.521^*^ | 2.272^*^ |
| *uvrA* | 1.013 | 1.062 |
| *yebG* | 1.126 | 1.578^*^ |
| cell membrane permeability |  |  |
| *bamB* | 1.879^*^ | 2.196^*^ |
| *exbB* | 1.262 | 2.694^*^ |
| *exbD* | 0.914 | 1.412^*^ |
| *mscS* | 1.132 | 1.924^*^ |
| *ompA* | 1.563^*^ | 1.386^**^ |
| *ompC* | 1.568^*^ | 1.450^*^ |
| *ompF* | 2.440^*^ | 2.361^*^ |
| *osmB* | 1.425^*^ | 2.462^*^ |
| *tolC* | 1.114 | 2.157^*^ |
| *waaA* | 2.039^*^ | 2.115^*^ |
| pilus generation |  |  |
| *fimC* | 1.564^**^ | 1.193^*^ |
| *fimD* | 2.654^**^ | 1.693^**^ |
| *fimG* | 1.614^*^ | 1.939^*^ |
| *fimH* | 1.526^*^ | 1.530^*^ |
| *fimI* | 1.108 | 1.087 |
| *yehB* | 1.746^*^ | 1.621^**^ |
| *yfcD* | 1.623^*^ | 2.237^*^ |
| ATP synthesis |  |  |
| *atpA* | 1.726** | 1.374^*^ |
| *atpB* | 1.432* | 2.122^*^ |
| *atpE* | 1.306^**^ | 2.529^**^ |
| *atpF* | 2.192^*^ | 1.054 |
| *atpG* | 2.572^*^ | 3.029^**^ |
| *atpH* | 1.130 | 2.244^**^ |
| T4SS |  |  |
| *T4CP* | 2.542^*^ | 2.597^*^ |
| *VirB1* | 2.492^**^ | 1.529^**^ |
| *VirB2* | 2.017^**^ | 2.452^**^ |
| T4SS | IBA | DS |
| *VirB3* | 2.109^*^ | 3.443^**^ |
| *VirB4* | 1.742^*^ | 1.623^*^ |
| *VirB5* | 2.693^*^ | 1.366^*^ |
| *VirB6* | 2.728^*^ | 1.723^*^ |
| *VirB7* | 1.953^*^ | 4.387^***^ |
| *VirB8* | 1.540^*^ | 2.447^**^ |
| *VirB9* | 2.079^*^ | 1.353^*^ |
| *VirB10* | 2.248^*^ | 1.893^*^ |
| *VirB11* | 3.704^***^ | 1.367^*^ |
| *VirD4* | 1.484^*^ | 1.995^**^ |

* *p* < 0.05, ** *p* < 0.01 and *** *p* < 0.001
